# Identification of a panel of MYC and Tip60 co-regulated genes functioning primarily in cell cycle and DNA replication

**DOI:** 10.18632/genesandcancer.175

**Published:** 2018-03

**Authors:** Ling-Jun Zhao, Paul M. Loewenstein, Maurice Green

**Affiliations:** ^1^ Department of Microbiology and Molecular Immunology, Saint Louis University School of Medicine, Doisy Research Center, St. Louis, Missouri, USA

**Keywords:** MYC, NuA4 complex, Tip60, p300, cancer

## Abstract

We recently reported that adenovirus E1A enhances MYC association with the NuA4/Tip60 histone acetyltransferase (HAT) complex to activate a panel of genes enriched for DNA replication and cell cycle. Genes from this panel are highly expressed in examined cancer cell lines when compared to normal fibroblasts. To further understand gene regulation in cancer by MYC and the NuA4 complex, we performed RNA-seq analysis of MD-MB231 breast cancer cells following knockdown of MYC or Tip60 - the HAT enzyme of the NuA4 complex. We identify here a panel of 424 genes, referred to as MYC-Tip60 co-regulated panel (MTcoR), that are dependent on both MYC and Tip60 for expression and likely co-regulated by MYC and the NuA4 complex. The MTcoR panel is most significantly enriched in genes involved in cell cycle and/or DNA replication. In contrast, genes repressed by shMYC but not by shTip60 (224 genes) have a low significance of enrichment in identifiable biological processes other than cell cycle and DNA replication. Genes repressed by shTip60 but not by shMYC (102 genes) have no significant identifiable gene enrichment. We propose that MYC cooperates with the NuA4 complex to activate the MTcoR panel of genes to promote DNA replication and cell cycle.

## INTRODUCTION

The proto-oncogene product MYC is a transcription factor over-expressed in many cancers [1-3]. It forms a leucine-zipper with MAX to bind promoters [4] and activate genes of multiple cellular pathways that govern cellular proliferation [1-3] and cancer prognosis [5]. Transcriptional regulation by MYC can involve histone acetyltransferase (HAT) complexes, such as the NuA4 complex and the GCN5 complex [6-9]. However, the roles of the HAT complexes during MYC function in cancer remains uncharacterized. The human NuA4 complex has up to 20 subunits [10] with a core HAT enzyme Tip60 to acetylate primarily histones H2 and H4 [11-13]. It is involved in chromatin remodeling, gene activation, and DNA damage repair [10, 14, 15]. MYC association with the NuA4 complex is inefficient in HeLa cells [16] and mouse embryonic stem cells [9], but may be enhanced in HeLa cells by the adenovirus E1A N-terminal domain [16, 17]. Enhanced MYC association with the NuA4 complex mediated by E1A results in activation of two panels of genes: the MNA4 panel (MYC NuA4) and the MNP300 panel (MYC NuA4 p300) [16]. Activation of the MNP300 panel requires E1A enhanced MYC association with the NuA4 complex as well as E1A targeting of p300/CBP [16]. The MNP300 panel is most significantly enriched in genes functioning in DNA replication and cell cycle. Importantly, selected genes from the MNP300 panel are highly expressed in the three examined cancer cell lines compared to normal fibroblasts [16], suggesting involvement of MYC association with the NuA4 complex in activation of the MNP300 panel in cancer cells.

Tip60 and TRRAP, both components of the NuA4 complex, have been identified as two of the six “hub genes” involved in signaling pathways of high importance to human cancers [18, 19], suggesting important roles of the NuA4 complex in cell proliferation and oncogenesis. Significantly, the HAT activity of Tip60 is functional only in the context of the NuA4 complex or NuA4-related “sub”-complexes [13, 14]. Proteomic analysis of Tip60 shows that it associates mainly with components of the NuA4 complex [9, 16, 20]. The yeast counterpart of Tip60, Esa1, is also found to exist only in the NuA4 complex [21]. In contrast, TRRAP, as a scaffold protein, helps assemble two additional HAT complexes: the GCN5 complex and the PCAF complex [10, 22]. Since MYC associates with TRRAP [7, 23-25], it is likely that MYC associates with all the three TRRAP-containing HAT complexes. Importantly, however, the adenovirus E1A oncoprotein N-terminal TRRAP-targeting domain, or the ET domain, targets the NuA4 complex [16] and enhances MYC association with the NuA4 complex [17], suggesting that the NuA4 complex may be critical for MYC and E1A function.

To further explore the function of MYC association with the NuA4 complex in cancer, we examined effects of shRNA-mediated knockdown of MYC and the Tip60 component of the NuA4 complex on gene expression in MD-MB231 human breast cancer cells. Our RNA-seq results suggest that MYC and Tip60 co-regulate the expression of a panel of genes, the MTcoR panel, that are critical for DNA replication and cell cycle.

## RESULTS

### Repression of selected genes by MYC and Tip60 knockdown

We previously identified the MNP300 panel of 352 genes that are activated in normal fibroblasts by the adenovirus E1A N-terminal domain through targeting p300 and the MYC-NuA4 complex [16]. Since the MNP300 panel of genes are enriched most significantly in genes involved in DNA replication and cell cycle, they may have important functions in cancer. Consistent with this possibility, selected genes from the MNP300 panel are over-expressed by 10-30 fold in three examined human cancer cell lines compared to normal fibroblasts [16]. To determine whether these genes are regulated by MYC and the NuA4 complex in human cancer cells, MYC and Tip60 (the core HAT enzyme in the NuA4 complex) were knocked down in MB231 human breast cancer cells by using two independent shRNA clones targeting different genomic regions of MYC or Tip60 (see Materials and Methods). Western blot analysis of cells expressing shMYC(1) or shMTC(2) showed efficient knockdown of MYC expression compared to control cells expressing shGFP (see Supplementary Figure 1, lanes 2 and 3). Western blot analysis of Tip60 with several commercial antibodies did not detect endogenous levels of Tip60 in cells likely due to lower expression of Tip60 and lower sensitivities of the antibodies.

**Figure 1:**
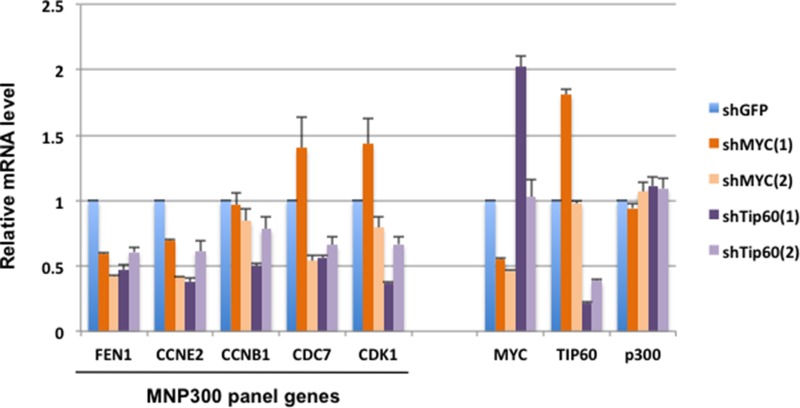
Repression of selected MNP300 panel genes by MYC and Tip60 shRNA MB231 cells transduced with individual shRNA clones for MYC and Tip60 were puromycin-selected and used for RT-qPCR analysis of genes of interest. Genes on the left are selected from the MNP300 panel for their roles in DNA replication and cell cycle. Results are average of the triplicate RNA samples that were also used for RNA-seq analysis. On the right: analysis of mRNA levels of MYC, Tip60, or p300 as a negative control. RT-qPCR primers for p300 and GAPDH (internal control) are listed in Supplementary Table 1, and other primers are as described earlier [16].

However, as expected, RT-qPCR analysis of cells expressing the shRNAs showed that the MYC shRNA clones efficiently inhibited MYC and the Tip60 shRNA clones efficiently inhibited Tip60 mRNA synthesis (Figure 1). Of interest, shMYC(1) and shTip60(1) mutually enhanced the mRNA synthesis of Tip60 and MYC, respectively (Figure 1) (see Discussion). As control, the level of p300 mRNA was not affected significantly by the expression of the shRNA clones (Figure 1). Among the MNP300 panel genes previously selected for analysis due to their roles in cell cycle and DNA replication [16], FEN1 and CCNE2 were most efficiently repressed by all four shRNA clones (Figure 1), and thus may be co-regulated by MYC and the NuA4 complex (represented by Tip60). Repression of CCNB1 was inefficient by all the shRNA clones, and repression of CDC7 and CDK1 appeared to be dependent on the shRNA clone. Specifically, shMYC(1), which enhanced Tip60 mRNA, also enhanced CDC7 and CDK1 mRNAs, whereas shMYC(2) repressed both genes. Since shMYC(1) appears to repress a lower number of genes than shMYC(2), it may serve as a more stringent selector of genes that are repressed specifically by MYC knockdown (see next). The co-repression of the selected MNP300 panel genes mediated by shMYC and shTip60 suggests that these shMYC and shTip60 clones are suitable for MYC and Tip60 knockdown and subsequent global analysis of their effects on gene repression.

### Identification of a MYC-Tip60 co-regulated (MTcoR) panel of genes by RNA-seq analysis following MYC and Tip60 knockdown

To examine global gene expression modulated by MYC and the NuA4 complex (represented by Tip60), we performed RNA-seq analysis with triplicate RNA samples prepared from MB231 cells expressing shMYC(1) and shMYC(2) to knockdown MYC, or shTip60(1) and shTip60(2) to knockdown Tip60. To identify genes that normally require MYC or Tip60 for expression, a list of differentially expressed protein-coding genes was generated by comparing their expression in shMYC - or shTip60 -expressing cells with control cells expressing shGFP. Subsequently, the gene list was filtered based on three criteria: i) repression by shMYC(1) and shTip60(1) is ≥ 30%, ii) adjusted P value is ≤ 0.05 for both shMYC(1) and shTip60(1), and iii) repression by shMYC(2) and shTip60(2) is ≥ 0. The more stringent requirements for shMYC(1) and shTip60(1) were due to the observation that these two shRNA clones enhance the expression of Tip60 and MYC, respectively (Figure 1), and may enhance gene expression through increased MYC or Tip60 expression. Thus, genes repressed by shMYC(1) and shTip60(1) with a high statistical significance (with adjusted P value ≤ 0.05) would have a high probability of being specifically repressed because of the knockdown of MYC and Tip60. These analyses resulted in a list of 424 genes (Supplementary Table 2), which is referred to as the MYC and Tip60 co-regulated panel, or MTcoR panel. Consistent with our criteria, FEN1 and CCNE2, which are repressed by all shMYC and shTip60 clones (Figure 1) are included in the MTcoR panel (Supplementary Table 2), whereas CDC7 and CDK1, which are enhanced by shMYC(1) (Figure 1), are excluded.

**Figure 2:**
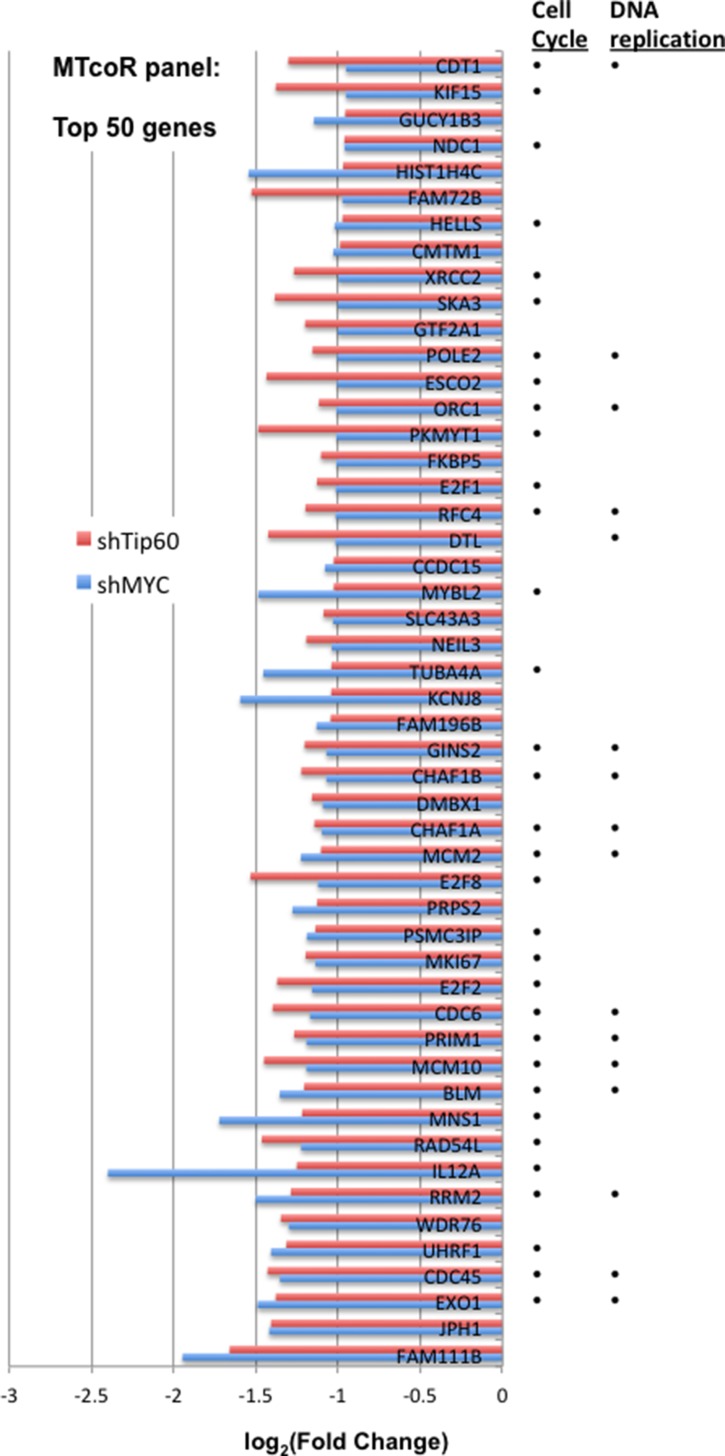
Repression of the top 50 genes from the MTcoR panel by shMYC and shTip60 MB231 cells were transduced in triplicate with shGFP (as control), shMYC(1), shMYC(2), shTip60(1) or shTip60(2) and selected with puromycin for two days. Total RNA was prepared and mRNA-seq analysis was performed after polyA-enrichment for mRNA. The MTcoR panel of genes (Table S2) that are co-regulated by MYC and Tip60 fit three criteria as described in the text. The MTcoR panel was sorted by the efficiency of co-repression by shMYC and shTip60, and the top 50 genes with the highest co-repression by shMYC and shTip60 are shown. Dots on the right: indicate function in either cell cycle or DNA replication.

The MTcoR panel was sorted by the efficiency of co-repression by shMYC and by shTip60, i.e., the extent of overlap between the repression efficiency of MYC knockdown (average of shMYC(1) and shMYC(2)) and the repression efficiency of Tip60 knockdown (average of shTip60(1) and shTip60(2)). The top 50 genes with the highest co-repression by shMYC and shTip60 are shown in Figure 2, and are good candidates for genes co-regulated by MYC and the NuA4 complex (represented by Tip60). As indicated in Figure 2, most of these genes are involved in cell cycle and DNA replication (see gene ontology analysis next).

### RT-qPCR analysis of gene repression by MYC and Tip60 knockdown

**Figure 3:**
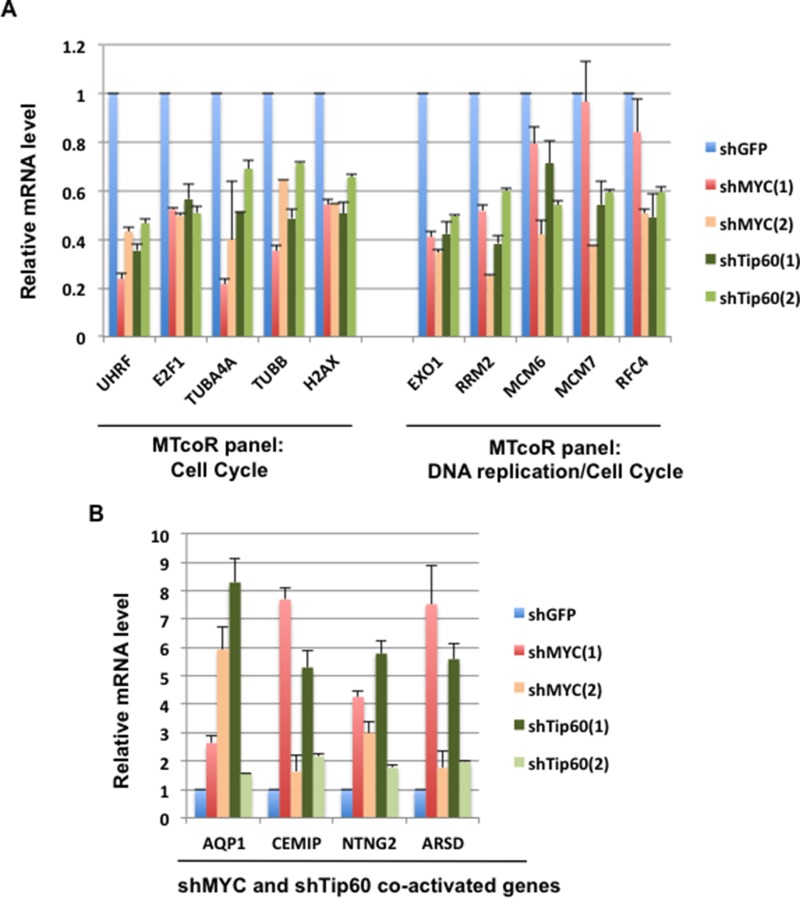
RT-qPCR analysis of selected genes from the MTcoR panel for repression by MYC and Tip60 knockdown **A.** MB231 cells were transduced with shRNA lentiviruses and selected with puromycin for two days before RNA preparation. Five genes from the MTcoR panel selected for their roles in cell cycle only (group on the left), and five genes in DNA replication/cell cycle (group on the right) are analyzed by RT-qPCR analysis. Results are average of three lentivirus transductions. RT-qPCR primers are listed in Supplementary Table 1. **B.** RT-qPCR analysis of four genes selected for their co-activation by shMYC and shTip60. Conditions are the same as in A.

To provide further evidence that the MTcoR panel of genes are co-regulated by MYC and Tip60, RT-qPCR analysis was performed for genes in the MTcoR panel that are involved in cell cycle and DNA replication as determined by gene ontology analysis described below. As shown in Figure 3A, the five genes that are involved in “cell cycle” are efficiently repressed by all shMYC and shTip60 clones. The five genes that are involved in both “DNA replication” and “cell cycle” (Figure 3A) are also repressed by shMYC and shTip60 clones, except that MCM7 and RFC4 are only marginally repressed by shMYC(1). Thus, although shMYC(1) and shMYC(2) both efficiently repress most of the MTcoR panel of genes, shMYC(1) appears to repress less number of genes than shMYC(2) as may be expected from the results of Figure 1. As a control for this analysis, four genes that were shown by RNA-seq analysis to be enhanced by shMYC and shTip60 are also shown to be efficiently activated during the RT-qPCR analysis (Figure 3B). We noted that shTip60(1) activated these genes much more efficiently than shTip60(2), possibly due to its enhancement of MYC expression (Figure 1). Similarly, to some extent, shMYC(1) also activated these genes more efficiently than shMYC(2). From these results, we conclude that the MTcoR panel of genes, identified by RNA-seq analysis, are also largely co-repressed by shMYC and shTip60 by RT-qPCR analysis, thereby strengthening the possibility that the MTcoR panel of genes are normally co-regulated by MYC and the NuA4 complex.

### Gene enrichment analysis of MTcoR panel for functional significance

**Figure 4:**
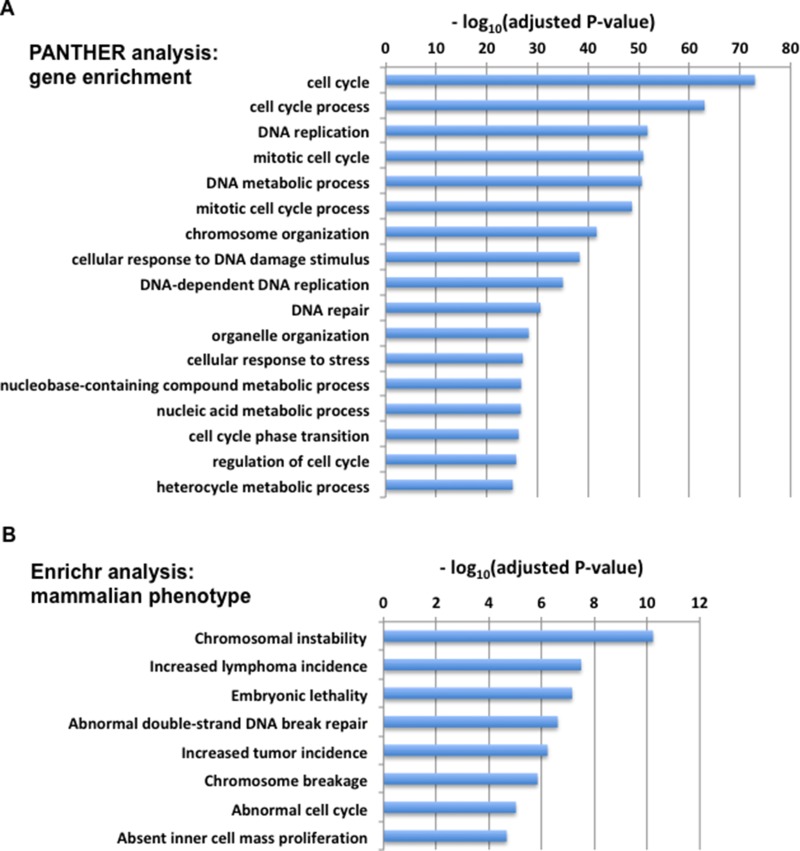
Gene ontology analysis and mammalian phenotype analysis for the MTcoR panel of genes **A.** PANTHER Gene Ontology analysis (PANTHER version 13). Biological processes with confidence defined by Fisher's Exact P value (with FDR - false discovery rate - multiple test correction) of < 1 × 10^−25^ are shown. Processes that appear to be redundant are at different levels of the gene classification hierarchy. **B.** Enrichr Mammalian phenotype analysis. The MGI (Mouse Genome Informatics) Mammalian Phenotype database contains experimental phenotypic changes due to targeted gene mutations in mouse. A high significance score (a low adjusted *P*-value) indicates enrichment of genes whose mutation has been shown to correlate with the phenotype in mouse models.

To evaluate the functional significance of the MTcoR panel of genes, these genes were subjected to gene ontology analysis using the PANTHER database of ∼21,000 genes with functional annotation [26, 27]. This analysis showed that the MTcoR panel is most significantly enriched in genes involved in cell cycle and DNA replication (Figure 4A). Of the 424 genes in the MTcoR panel, 164 are involved in cell cycle (false discovery rate (FDR)-adjusted P value of 7.69 × 10^−78^), and 70 are involved in DNA replication and mostly overlap with cell cycle genes (FDR-adjusted P value of 3.65 × 10^−56^). MTcoR panel genes involved in cell cycle represent ∼ 6-fold enrichment compared to the database, whereas those involved in DNA replication represent ∼16-fold enrichment. The highly significant P values also indicate that cell cycle and DNA replication are the main function of the MTcoR panel of genes. Importantly, the MTcoR panel of genes are also enriched in biological processes that seem to facilitate DNA replication and cell cycle, such as “chromosome organization” and “Nucleic acid metabolic processes”.

In contrast, similar analysis revealed that genes repressed by shMYC (by ≥ 30%) but not by shTip60 (repression by shTip60 ≤ 10%) (224 genes) have a low significance of gene enrichment in several identifiable biological processes excluding cell cycle and DNA replication (data not shown). Genes repressed by shTip60 but not by shMYC (102 genes) have no identifiable gene enrichment (data not shown). Thus, gene regulatory functions of MYC and the NuA4 complex (represented by Tip60) may be exerted predominantly by their cooperation with each other in activation of the MTcoR panel of genes that primarily function in cell cycle and DNA replication. In contrast, genes that are regulated by MYC or the NuA4 complex separately may affect diverse cellular processes. We also noted that the top 424 genes co-activated by shMYC and by shTip60 (by ≥ 47%) have no identifiable gene enrichment (not shown), suggesting that these genes may be normally suppressed by MYC and the NuA4 complex and function in multiple cellular processes. These gene ontology analyses indicate an overall pattern of gene regulation by MYC and the NuA4 complex, i.e., only genes whose expression is normally activated cooperatively by MYC and the NuA4 complex have a predominant function in cell cycle and DNA replication.

The MTcoR panel of genes are also subjected to Enrichr analysis for mammalian phenotypes (http://amp.pharm.mssm.edu/Enrichr/) [28, 29]. This analysis is based on the Mouse Genome Informatics (MGI) database for mammalian phenotypes which are derived from mouse models through targeted gene mutations, such as knockout of the H2AX gene [30] or mutations that cripple the functional domain of Exo1 [31]. Both H2AX and Exo1 are identified in the MTcoR panel (Figure 3). Importantly, Enrichr analysis of the MTcoR panel (Figure 4B) revealed that it has the most significant enrichment in genes whose mutation may be associated with chromosomal instability (14 genes from the MTcoR panel) and increased lymphoma incidence (16 genes from the MTcoR panel) (Figure 4B). In the MGI database, 658 genotypes are correlated with an increased lymphoma incidence, and 212 genotypes with chromosomal instability (http://www.informatics.jax.org/). These results are consistent with the conclusion from the PANTHER gene enrichment analysis that the MTcoR panel of genes are involved in cell cycle regulation and DNA replication.

### Repression of selected MTcoR panel of genes in other cancer cell lines by MYC and Tip60 shRNA

**Figure 5:**
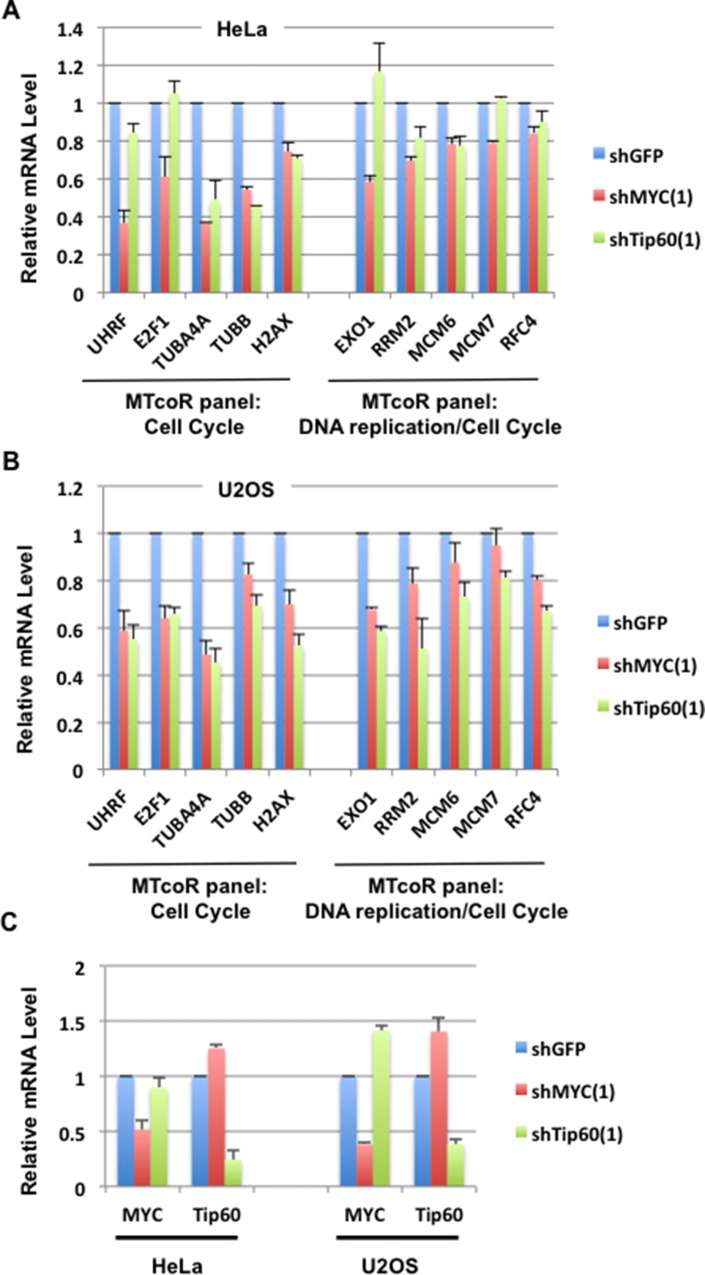
Examination of effects of shMYC or shTip60 on expression of selected MTcoR panel genes in HeLa and U2OS cells **A.** RT-qPCR analysis of selected MTcoR panel of genes in HeLa cells after shRNA mediated knockdown of MYC or Tip60. Since both shRNAs caused significant changes in expression of the endogenous control GAPDH, RT-qPCR analysis was standardized by using equal quantities of input RNA. Results are the average of two lentiviral transductions. **B.** RT-qPCR analysis of selected MTcoR panel of genes in U2OS cells. Conditions are the same as in A. **C.** RT-qPCR analysis of MYC and Tip60 in HeLa and U2OS cells.

To determine whether the MTcoR panel of genes are regulated by MYC and Tip60 also in other cancer cell lines, shMYC(1) and shTip60(1) were expressed in HeLa and U2OS cells, and then expression of selected genes (same as in Figure 3A) was analyzed. As shown, most of the selected genes are inhibited by both shMYC(1) and shTip60(1) in both cell lines (Figure 5A and 5B). In particular, TBUA4A, TUBB, and H2AX appear to have the highest degree of co-dependence on MYC and Tip60 for expression in both cell lines. In contrast, knockdown of MYC repressed UHRF, E2F1, and Exo1 in both U2OS and HeLa cells, whereas Tip60 knockdown repressed these genes in U2OS cells but not in HeLa cells (see Discussion). Thus, different cancer cell lines may have a common set of MTcoR genes that are co-regulated by MYC and the NuA4 complex (represented by Tip60). Other MTcoR genes may be co-regulated by MYC and the NuA4 complex in a cell type-specific manner. Additionally, shMYC(1) and shTip60(1) appear to significantly enhance expression of Tip60 and MYC, respectively, in U2OS cells, but only marginally in HeLa cells (Figure 5C).

## DISCUSSION

**Figure 6:**
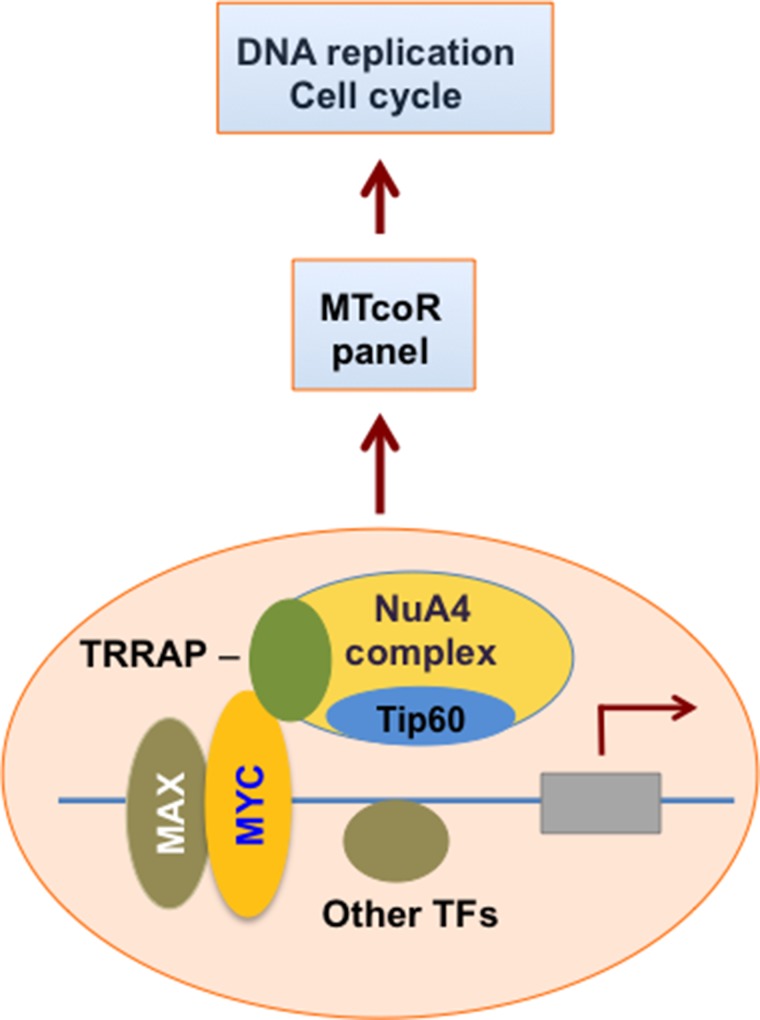
A model for the function of the MYC-NuA4 complex MYC recruits the NuA4 complex to specific gene promoters, leading to acetylation of promoter histones and activation of the MTcoR panel of genes, possibly in cooperation with other transcription factors (TF). Expression of the MTcoR panel of genes promotes DNA replication and cell cycle. Interference of the normal function of the MYC-NuA4 complex would lead to dysfunction of cell cycle and potential transformation or cell death.

MYC and Tip60 - the essential HAT enzyme of the NuA4 complex, play critical roles in cellular proliferation and death. Cooperativity between MYC and Tip60 in promoter regulation has been observed in mouse embryonic stem cells [32]. Tip60, possibly in the context of the NuA4 complex, may directly acetylate MYC [33]. Our previous studies with adenovirus E1A N-terminal domain helped identify an MNP300 panel of genes that are activated by E1A targeting of both the MYC-NuA4 complex and p300/CBP [16]. The MNP300 panel shares 54 genes with the MTcoR panel identified in this study, including FEN1 and CCNE2, which are repressed by both shMYC and shTip60 (Figure 1). Gene ontology analysis shows that these overlapping genes are also predominantly enriched in genes involved in cell cycle and DNA replication (Table 1). In fact, 39 genes (72%) of the 54 genes are involved in cell cycle regulation, and 20 of these 39 genes are also involved in DNA replication (Table 1). In contrast, 164 genes of the 424 genes in the MTcoR panel, representing ∼39%, are involved in cell cycle. These results support the thesis that E1A N-terminal domain targets the MYC-NuA4 complex to up-regulate genes involved in cell cycle/DNA replication probably to promote conditions suitable for adenovirus replication [16]. Under appropriate conditions and lack of functional check points these changes in gene expression may lead to cellular transformation. Importantly, in the top 50 genes from MTcoR panel (Figure 2), 33 genes - representing 66% - are involved in cell cycle, suggesting that genes with a higher degree of co-regulation by MYC and the NuA4 complex have a higher probability of involvement in cell cycle. We propose that MYC and the NuA4 complex may cooperate to activate the MTcoR panel of genes that function mainly in cell cycle regulation and DNA replication (Figure 6) and may play major roles in cancer development. Conceivably, this cooperativity could be achieved by the recruitment of a MYC-NuA4 complex to the MTcoR gene promoters, or by recruitment of MYC and the NuA4 complex separately.

**Table 1:** Genes overlapping between the MTcoR panel and the MNP300 panel and involved in cell cycle

#	Gene name	Full name	Functional class
1	GINS2*	DNA REPLICATION COMPLEX GINS PROTEIN PSF2	nuclease
2	TICRR	TRESLIN	-
3	EZH2	HISTONE-LYSINE N-METHYLTRANSFERASE EZH2	-
4	CCNE2	G1/S-SPECIFIC CYCLIN-E2	kinase activator
5	DDIAS	DNA DAMAGE-INDUCED APOPTOSIS SUPPRESSOR PROTEIN	-
6	ZWINT	ZW10 INTERACTOR	-
7	HAUS5	HAUS AUGMIN-LIKE COMPLEX SUBUNIT 5	-
8	CHAF1B	CHROMATIN ASSEMBLY FACTOR 1 SUBUNIT B	chromatin/chromatin-binding protein
9	CDC45	CELL DIVISION CONTROL PROTEIN 45 HOMOLOG	replication origin binding protein
10	NCAPD3	CONDENSIN-2 COMPLEX SUBUNIT D3	nucleic acid binding
11	H2AFX	HISTONE H2AX	histone
12	PLK1	SERINE/THREONINE-PROTEIN KINASE PLK1	-
13	MNS1	MEIOSIS-SPECIFIC NUCLEAR STRUCTURAL PROTEIN 1	structural protein
14	RRM2	RIBONUCLEOSIDE-DIPHOSPHATE REDUCTASE SUBUNIT M2	reductase
15	TYMS	THYMIDYLATE SYNTHASE	methyltransferase
16	DSCC1	SISTER CHROMATID COHESION PROTEIN DCC1	replication origin binding protein
17	E2F1	TRANSCRIPTION FACTOR E2F1	transcription factor
18	RFC5	REPLICATION FACTOR C SUBUNIT 5	DNA-directed DNA polymerase
19	FANCA	FANCONI ANEMIA GROUP A PROTEIN	-
20	DCLRE1A	DNA CROSS-LINK REPAIR 1A PROTEIN	-
21	FEN1	FLAP ENDONUCLEASE 1	endo/exodeoxyribonuclease
22	RFC2	REPLICATION FACTOR C SUBUNIT 2	DNA-directed DNA polymerase
23	CDC6	CELL DIVISION CONTROL PROTEIN 6 HOMOLOG	-
24	DNA2	ATP-DEPENDENT HELICASE/NUCLEASE DNA2	DNA helicase, RNA helicase
25	EXO1	EXONUCLEASE 1	endo/exodeoxyribonuclease
26	MYBL2	MYB-RELATED PROTEIN B	-
27	HELLS	LYMPHOID-SPECIFIC HELICASE	-
28	GINS1	DNA REPLICATION COMPLEX GINS PROTEIN PSF1	nucleic acid binding
29	INCENP	INNER CENTROMERE PROTEIN	-
30	LIG1	DNA LIGASE 1	DNA ligase
31	RBBP8	DNA ENDONUCLEASE RBBP8	transcription cofactor
32	YEATS4	YEATS DOMAIN-CONTAINING PROTEIN 4	transcription factor
33	DONSON	PROTEIN DOWNSTREAM NEIGHBOR OF SON)	-
34	BLM	BLOOM SYNDROME PROTEIN	-
35	KIF18B	KINESIN-LIKE PROTEIN KIF18B	microtubule binding motor protein
36	KIF15	KINESIN-LIKE PROTEIN KIF15	microtubule binding motor protein
37	CDT1	DNA REPLICATION FACTOR CDT1	-
38	CLSPN	CLASPIN	-
39	MAD2L1	MITOTIC SPINDLE ASSEMBLY CHECKPOINT PROTEIN MAD2A	-

* genes highlighted in pink are also involved in DNA replication.

Additional analysis of the RNA-seq result by sorting for genes repressed by shMYC by ≥ 30% and not repressed by shTip60 (defined as ≤ 10% repression) revealed 224 genes that are enriched for a few cellular processes with FDR-adjusted P values of ≥ 7.89E-07 (not shown), as compared to the MTcoR panel's enrichment in cell cycle with an FDR-adjusted P value of 7.6989E-78. No enrichment in cell cycle or DNA replication can be identified in these MYC-dependent and Tip60-indepent genes. Thus, MYC may target transcription factors different from the NuA4 complex, such as the GCN5 HAT complex [6, 7], for activation of these genes. In contrast, sorting for genes repressed by shTip60 by ≥ 30% and not repressed by shMYC revealed 102 genes that have no enrichment for any identifiable biological processes, suggesting that Tip60 functions predominantly together with MYC, in the form of a MYC-NuA4 complex. This possibility is consistent with the proteomic analysis showing that Tip60 exists predominantly in the context of the NuA4 complex [9, 13, 16, 20]. Nevertheless, we cannot exclude the possibility that some of the MTcoR panel of genes are regulated by MYC and by the NuA4 complex on the same promoter in the absence of a direct interaction.

Since a high fraction of human cancers have MYC over-expression, MYC is an attractive cancer target [34, 35]. For example, p53 and MYC are found to co-regulate genes aberrantly expressed in leukemic stem cells, and targeting p53 and MYC simultaneously selectively eliminates leukemic stem cells [36]. The primary function of the MTcoR panel of genes in DNA replication and cell cycle suggests that the MTcoR panel may be of potential importance for cancer therapies targeting MYC and the NuA4 complex. Comparison of effects of MYC and Tip60 knockdown on MTcoR panel gene expression in different cell lines (Figure 3 and Figure 5) suggests that there are genes co-regulated by MYC and the NuA4 complex in many cancer cell lines, as well as genes co-regulated by MYC and the NuA4 complex in a cell type-specific manner. Thus, identification of a common set of MTcoR panel genes that are co-regulated by MYC and the NuA4 complex in most cancer lines may be valuable in understanding cancer development. Significantly, shMYC(1) and shTip60(1) mutually enhance expression of Tip60 and MYC in MB231 (Figure 1) and U2OS cells (Figure 5C). These shRNA clones have been used in various studies to explore gene regulatory pathways in cancer [37, 38]. Although the mechanism remains unclear, it may reflect a cellular mechanism to compensate for the loss of MYC or a functional NuA4 complex (due to lack of Tip60), by increasing the expression of MYC or Tip60, respectively. This is consistent with the important functions of the MTcoR panel of genes in DNA replication and cell cycle. Nevertheless, most of the MTcoR panel of genes remain susceptible to repression by shMYC(1) and shTip60(1), and increasing MYC or Tip60 synthesis alone fails to normalize the expression of the MTcoR panel of genes. Thus, the MTcoR panel of genes may be targeted for cancer therapy, alone or in combination with targeting of MYC and the NuA4 complex.

## MATERIALS AND METHODS

### Cell culture and lentiviral transduction

Human breast cancer cell line MD-MB231 (ATCC), HEK 293T (Invitrogen), HeLa and U2OS (osteosarcoma, ATCC) were cultured in DMEM (Life Technologies) supplemented with 10% fetal bovine serum and 50 U/ml of Pen/Strep. Lentiviruses were generated by transfection in 293T cells as described previously [17]. Cells in 100 mm dishes were transduced with shRNA lentiviruses overnight, selected with puromycin (1 μg/ml) for two days, and used for RNA preparation as described previously[16]. Alternatively, MB231 cells were plated in 6-well dishes, transduced and drug-selected the same way, and then lysed for Western blot analysis. All shRNA lentiviral clones are from Sigma and target different genomic regions of MYC and Tip60: shMYC(1) - TRCN0000039642 (target sequence: CCTGAGACAGATCAGCAACAA), shMYC(2) - TRCN0000312581 (target sequence:CCAGAGGAGGAACGAGCTAAA); shTip60(1) - TRCN0000020315 (target sequence: CCTCAATCTCATCAACTACTA), shTip60(2) - TRCN0000020317 (target sequence: CCTCCTATCCTATCGAAGCTA).

### RT-qPCR analysis and RNA-seq analysis

RNA was prepared in triplicates as described previously [16], and subjected to PolyA selection and RNA-seq analysis at the Washington University Genome Technology Access Center as described earlier [16]. RT-qPCR analysis was as described previously [16].

### Gene enrichment analysis

PANTHER (version 13) gene enrichment analysis [26, 27] was performed online (http://www.geneontology.org). Biological processes with False Discovery Rate (FDR) corrected P values of smaller than the defined value are shown in Figure 4. Ontologies - MGI (Mouse Genome Informatics) Mammalian phenotype analysis using ENRICHR was performed online (http://amp.pharm.mssm.edu/Enrichr/). The MGI database contains annotated phenotypes induced by targeted gene mutation/inactivation in mouse [28, 29].

## SUPPLEMENTARY MATERIAL FIGURE AND TABLES


