# Supplementary material for: Identification of a panel of MYC and Tip60 co-regulated genes functioning primarily in cell cycle and DNA replication
Source: Genes Cancer. 2018 Mar;9(3-4):101–13. doi: 10.18632/genesandcancer.175 (PMC6086004; doi:10.18632/genesandcancer.175)
Supplement: Supplementary file 1 [file ganc-09-101-s001.pdf]

Identification of a panel of MYC and Tip60 co-regulated genes functioning primarily in cell cycle and DNA replication – Zhao et al

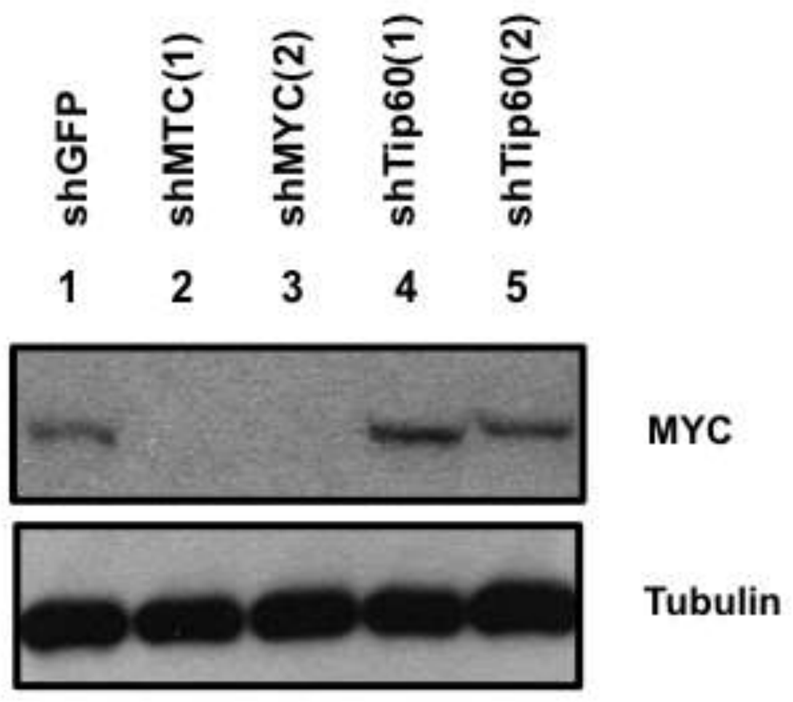

Figure S1: Knockdown of MYC by shMYC(1) and shMYC(2) in MB231 cells.

Human breast cancer cells (MB231) were transduced with lentiviruses expressing shMYC or shTip60 (2 clones each), selected with puromycin and examined by Western blot analysis with MYC antibody (**AF3696**, R&D Systems). Both shMYC(1) (lane 2) and shMYC(2) (lane 3) strongly reduced MYC protein level compared to cells expressing shGFP (lane 1). Compared to RT-qPCR analysis (Figure 1), Western blot appeared to show a more drastic reduction in MYC levels by shMYC(1) and shMYC(2). However, this could be due to a lower detection sensitivity of Western blot. Consistent with RT-qPCR results (Figure 1), shTip60(1) appeared to enhance MYC level (lane 4). Western blot analysis of the same samples with various Tip60 antibodies did not detect Tip60, possibly due to the detection of only recombinantly expressed Tip60 by the commercial antibodies (see for example, Tip60 antibody GTX112197 from GeneTex). The endogenous level of the yeast counterpart of the human Tip60, ESA1, also could not be detected by Western blot [1].

1. Allard S, Utley RT, Savard J, Clarke A, Grant P, Brandl CJ, Pillus L, Workman JL, and Cote J. NuA4, an essential transcription adaptor/histone H4 acetyltransferase complex containing Esa1p and the ATM-related cofactor Tra1p. EMBO J. 1999;18(18):5108-19.

Table S1: PCR primers (listed according to order of appearance in paper)

GAPDH-F*	CCATGAGAAGTATGACAACAGCCT
GAPDH-R**	TGAGTCCTTCCACGATACCAAAGT
P300-F	AGCCAAGCGGCCTAAACTCTCATC
P300-R	TCACCACCATTGGTTAGTCCCAAT
UHRF1-F	GCCATACCCTCTTCGACTACG
UHRF1-R	GCCCCAATTCCGTCTCATCC
E2F1-F	ACGTGACGTGTCAGGACCT
E2F1-R	GATCGGGCCTTGTTTGCTCTT
TUBA4A-F	CGGCTCTCTGTTGACTATGGC
TUBA4A-R	GGCGCTCGATGTCTAGGTT
TUBB-F	TGGACTCTGTTCGCTCAGGT
TUBB-R	TGCCTCCTTCCGTACCACAT
H2AX-F	AAGAAGACGCGAATCATCCC
H2AX-R	CTGGATGTTGGGCAGGAC
EXO1-F	CCTCGTGGCTCCCTATGAAG
EXO1-R	AGGAGATCCGAGTCCTCTGTAA
RRM2-F	GTGGAGCGATTTAGCCAAGAA
RRM2-R	CACAAGGCATCGTTTCAATGG
MCM6-F	GAGGAACTGATTCGTCCTGAGA
MCM6-R	CAAGGCCCGACACAGGTAAG
MCM7-F	GCCTGTGGGAAATATCCCTCG
MCM7-R	GTACCACCTGTCGGAACCC
RFC4-F	TTGGGCCTGAACTTTTCCGAT
RFC4-R	AGCGACTTCCTGACACAGTTA
AQP1-F	TAACCCTGCTCGGTCCTTTG
AQP1-R	AGTCGTAGATGAGTACAGCCAG
CEMIP-F	GAACCCGGCACATCCTGATT
CEMIP-R	GATCCGGCTGAATACCTTCATC
NTG2-F	GCGCCTGAAGGACTACGTC
NTG2-R	CGTTGCTGCATAGGTAGGGAT
ARSD-F	TTCCTTCAGATCAGGCATGGA
ARSD-R	ACCCTGGTGCCATTTTCCTAT

*F: forward

**R: reverse

Table S2: MTcoR panel of genes

entrezgene	external_gen	description	shMYC(1)_logFC	shMYC(2)_logFC	shTip60(1)_logFC	shTip60(2)_logFC	Ave_shMYC_logI	Ave_shTip60_lo	Abs(Overlap)_logFC**
374393	FAM111B*	family with sequence similarity 111 member B [Source:HGNC Sym	-2.20	-1.69	-2.08	-1.24	-1.95	-1.66	1.66
56704	JPH1	junctophilin 1 [Source:HGNC Symbol;Acc:HGNC:14201]	-0.89	-1.95	-1.07	-1.75	-1.42	-1.41	1.41
9156	EXO1	exonuclease 1 [Source:HGNC Symbol;Acc:HGNC:3511]	-1.49	-1.49	-1.73	-1.02	-1.49	-1.38	1.38
8318	CDC45	cell division cycle 45 [Source:HGNC Symbol;Acc:HGNC:1739]	-1.17	-1.53	-1.87	-0.98	-1.35	-1.43	1.35
29128	UHRF1	ubiquitin like with PHD and ring finger domains 1 [Source:HGNC S	-2.09	-0.73	-1.78	-0.85	-1.41	-1.31	1.31
79968	WDR76	WD repeat domain 76 [Source:HGNC Symbol;Acc:HGNC:25773]	-1.45	-1.15	-1.71	-0.98	-1.30	-1.35	1.30
6241	RRM2	ribonucleotide reductase regulatory subunit M2 [Source:HGNC Sy	-1.18	-1.82	-1.89	-0.69	-1.50	-1.29	1.29
3592	IL12A	interleukin 12A [Source:HGNC Symbol;Acc:HGNC:5969]	-2.32	-2.48	-1.68	-0.82	-2.40	-1.25	1.25
8438	RAD54L	RAD54 like [Source:HGNC Symbol;Acc:HGNC:9826]	-1.40	-1.05	-1.92	-1.01	-1.22	-1.46	1.22
55329	MNS1	meiosis specific nuclear structural 1 [Source:HGNC Symbol;Acc:HG	-1.79	-1.66	-1.27	-1.17	-1.72	-1.22	1.22
641	BLM	Bloom syndrome RecQ like helicase [Source:HGNC Symbol;Acc:HG	-1.39	-1.32	-1.56	-0.85	-1.36	-1.21	1.21
55388	MCM10	minichromosome maintenance 10 replication initiation factor [So	-1.00	-1.38	-1.94	-0.97	-1.19	-1.45	1.19
5557	PRIM1	DNA primase subunit 1 [Source:HGNC Symbol;Acc:HGNC:9369]	-1.00	-1.38	-1.46	-1.07	-1.19	-1.27	1.19
990	CDC6	cell division cycle 6 [Source:HGNC Symbol;Acc:HGNC:1744]	-1.15	-1.19	-1.89	-0.91	-1.17	-1.40	1.17
1870	E2F2	E2F transcription factor 2 [Source:HGNC Symbol;Acc:HGNC:3114]	-1.44	-0.88	-1.78	-0.96	-1.16	-1.37	1.16
4288	MKI67	marker of proliferation Ki-67 [Source:HGNC Symbol;Acc:HGNC:71	-1.73	-0.54	-1.65	-0.75	-1.14	-1.20	1.14
29893	PSMC3IP	PSMC3 interacting protein [Source:HGNC Symbol;Acc:HGNC:1792	-1.23	-1.16	-1.45	-0.83	-1.19	-1.14	1.14
5634	PRPS2	phosphoribosyl pyrophosphate synthetase 2 [Source:HGNC Symb	-1.69	-0.86	-1.94	-0.32	-1.28	-1.13	1.13
79733	E2F8	E2F transcription factor 8 [Source:HGNC Symbol;Acc:HGNC:24727	-1.40	-0.85	-1.96	-1.11	-1.12	-1.53	1.12
4171	MCM2	minichromosome maintenance complex component 2 [Source:HG	-1.31	-1.14	-1.36	-0.85	-1.23	-1.10	1.10
10036	CHAF1A	chromatin assembly factor 1 subunit A [Source:HGNC Symbol;Acc	-1.17	-1.03	-1.46	-0.83	-1.10	-1.14	1.10
127343	DMBX1	diencephalon/mesencephalon homeobox 1 [Source:HGNC Symbo	-1.52	-0.67	-1.64	-0.68	-1.09	-1.16	1.09
8208	CHAF1B	chromatin assembly factor 1 subunit B [Source:HGNC Symbol;Acc	-1.02	-1.12	-1.34	-1.10	-1.07	-1.22	1.07
51659	GINS2	GINS complex subunit 2 [Source:HGNC Symbol;Acc:HGNC:24575]	-0.93	-1.21	-1.55	-0.86	-1.07	-1.20	1.07
100131897	FAM196B	family with sequence similarity 196 member B [Source:HGNC Sym	-1.38	-0.88	-1.27	-0.82	-1.13	-1.04	1.04
3764	KCNJ8	potassium voltage-gated channel subfamily J member 8 [Source:H	-1.79	-1.40	-1.24	-0.85	-1.60	-1.04	1.04
7277	TUBA4A	tubulin alpha 4a [Source:HGNC Symbol;Acc:HGNC:12407]	-2.45	-0.46	-1.49	-0.59	-1.46	-1.04	1.04
55247	NEIL3	nei like DNA glycosylase 3 [Source:HGNC Symbol;Acc:HGNC:2457	-1.26	-0.82	-1.73	-0.65	-1.04	-1.19	1.04
29015	SLC43A3	solute carrier family 43 member 3 [Source:HGNC Symbol;Acc:HGN	-1.77	-0.29	-1.46	-0.71	-1.03	-1.09	1.03
4605	MYBL2	MYB proto-oncogene like 2 [Source:HGNC Symbol;Acc:HGNC:754	-1.11	-1.86	-1.30	-0.75	-1.49	-1.03	1.03
80071	CCDC15	coiled-coil domain containing 15 [Source:HGNC Symbol;Acc:HGN	-1.25	-0.91	-1.21	-0.84	-1.08	-1.03	1.03
51514	DTL	denticleless E3 ubiquitin protein ligase homolog [Source:HGNC Sy	-1.18	-0.85	-1.98	-0.87	-1.02	-1.43	1.02
5984	RFC4	replication factor C subunit 4 [Source:HGNC Symbol;Acc:HGNC:99	-1.10	-0.93	-1.52	-0.87	-1.01	-1.20	1.01
1869	E2F1	E2F transcription factor 1 [Source:HGNC Symbol;Acc:HGNC:3113]	-1.27	-0.76	-1.32	-0.94	-1.01	-1.13	1.01
2289	FKBP5	FK506 binding protein 5 [Source:HGNC Symbol;Acc:HGNC:3721]	-1.10	-0.92	-1.35	-0.85	-1.01	-1.10	1.01
9088	PKMYT1	protein kinase, membrane associated tyrosine/threonine 1 [Sourc	-1.05	-0.97	-2.10	-0.87	-1.01	-1.49	1.01
4998	ORC1	origin recognition complex subunit 1 [Source:HGNC Symbol;Acc:H	-1.02	-0.99	-1.40	-0.83	-1.01	-1.12	1.01
157570	ESCO2	establishment of sister chromatid cohesion N-acetyltransferase 2	-0.86	-1.15	-1.98	-0.90	-1.01	-1.44	1.01
5427	POLE2	DNA polymerase epsilon 2, accessory subunit [Source:HGNC Symb	-0.95	-1.06	-1.45	-0.86	-1.01	-1.16	1.01
2957	GTF2A1	general transcription factor IIA subunit 1 [Source:HGNC Symbol;A	-1.60	-0.40	-2.23	-0.18	-1.00	-1.20	1.00
221150	SKA3	spindle and kinetochore associated complex subunit 3 [Source:HG	-1.09	-0.91	-1.79	-0.98	-1.00	-1.39	1.00
7516	XRCC2	X-ray repair cross complementing 2 [Source:HGNC Symbol;Acc:HG	-1.00	-1.00	-1.78	-0.76	-1.00	-1.27	1.00
113540	CMTM1	CKLF like MARVEL transmembrane domain containing 1 [Source:H	-1.76	-0.30	-1.36	-0.61	-1.03	-0.99	0.99
3070	HELLS	helicase, lymphoid specific [Source:HGNC Symbol;Acc:HGNC:4861	-1.12	-0.92	-1.23	-0.71	-1.02	-0.97	0.97
653820	FAM72B	family with sequence similarity 72 member B [Source:HGNC Symb	-0.94	-1.00	-2.18	-0.87	-0.97	-1.53	0.97
8364	HIST1H4C	histone cluster 1 H4 family member c [Source:HGNC Symbol;Acc:H	-1.57	-1.52	-1.53	-0.41	-1.54	-0.97	0.97
55706	NDC1	NDC1 transmembrane nucleoporin [Source:HGNC Symbol;Acc:HG	-1.59	-0.33	-1.03	-0.89	-0.96	-0.96	0.96
2983	GUCY1B3	guanylate cyclase 1 soluble subunit beta [Source:HGNC Symbol;A	-1.31	-0.99	-1.43	-0.48	-1.15	-0.96	0.96
56992	KIF15	kinesin family member 15 [Source:HGNC Symbol;Acc:HGNC:1727	-1.15	-0.76	-2.09	-0.66	-0.95	-1.38	0.95
81620	CDT1	chromatin licensing and DNA replication factor 1 [Source:HGNC Sy	-1.13	-0.77	-1.70	-0.90	-0.95	-1.30	0.95
401541	CENPP	centromere protein P [Source:HGNC Symbol;Acc:HGNC:32933]	-0.76	-1.14	-1.21	-0.88	-0.95	-1.04	0.95
63967	CLSPN	claspin [Source:HGNC Symbol;Acc:HGNC:19715]	-1.18	-0.72	-1.55	-0.85	-0.95	-1.20	0.95
2305	FOXM1	forkhead box M1 [Source:HGNC Symbol;Acc:HGNC:3818]	-1.30	-0.58	-1.40	-0.77	-0.94	-1.09	0.94
3014	H2AFX	H2A histone family member X [Source:HGNC Symbol;Acc:HGNC:47	-1.20	-0.67	-1.54	-0.74	-0.93	-1.14	0.93
7015	TERT	telomerase reverse transcriptase [Source:HGNC Symbol;Acc:HGNC	-1.46	-1.45	-1.28	-0.58	-1.46	-0.93	0.93
83879	CDCA7	cell division cycle associated 7 [Source:HGNC Symbol;Acc:HGNC:14	-1.24	-0.87	-1.09	-0.77	-1.05	-0.93	0.93
11169	WDHD1	WD repeat and HMG-box DNA binding protein 1 [Source:HGNC Syn	-1.07	-0.79	-1.44	-0.72	-0.93	-1.08	0.93
91860	CALML4	calmodulin like 4 [Source:HGNC Symbol;Acc:HGNC:18445]	-1.04	-0.80	-1.11	-0.86	-0.92	-0.99	0.92
4900	NRGN	neurogranin [Source:HGNC Symbol;Acc:HGNC:8000]	-1.29	-0.55	-1.13	-0.75	-0.92	-0.94	0.92
100288413	ERVMER34-1	endogenous retrovirus group MER34 member 1, envelope [Source:	-0.92	-0.92	-0.87	-1.11	-0.92	-0.99	0.92
9134	CCNE2	cyclin E2 [Source:HGNC Symbol;Acc:HGNC:1590]	-0.76	-1.08	-1.36	-0.60	-0.92	-0.98	0.92
162681	C18orf54	chromosome 18 open reading frame 54 [Source:HGNC Symbol;Acc:	-1.03	-0.80	-1.28	-0.64	-0.92	-0.96	0.92
6949	TCOF1	treacle ribosome biogenesis factor 1 [Source:HGNC Symbol;Acc:HG	-1.79	-1.67	-1.09	-0.74	-1.73	-0.92	0.92
2237	FEN1	flap structure-specific endonuclease 1 [Source:HGNC Symbol;Acc:H	-1.16	-0.99	-1.18	-0.65	-1.07	-0.92	0.92
84904	ARHGEF39	Rho guanine nucleotide exchange factor 39 [Source:HGNC Symbol;	-0.89	-0.93	-1.68	-0.54	-0.91	-1.11	0.91
771	CA12	carbonic anhydrase 12 [Source:HGNC Symbol;Acc:HGNC:1371]	-3.04	-0.09	-1.78	-0.04	-1.56	-0.91	0.91
137994	LETM2	leucine zipper and EF-hand containing transmembrane protein 2 [S	-1.33	-0.74	-1.30	-0.52	-1.03	-0.91	0.91
2707	GJB3	gap junction protein beta 3 [Source:HGNC Symbol;Acc:HGNC:4285]	-1.53	-0.29	-1.40	-0.47	-0.91	-0.93	0.91
64785	GINS3	GINS complex subunit 3 [Source:HGNC Symbol;Acc:HGNC:25851]	-0.83	-0.98	-1.35	-0.83	-0.91	-1.09	0.91
2123	EVI2A	ecotropic viral integration site 2A [Source:HGNC Symbol;Acc:HGNC	-2.03	-0.07	-1.72	-0.08	-1.05	-0.90	0.90
27346	TMEM97	transmembrane protein 97 [Source:HGNC Symbol;Acc:HGNC:2810	-1.72	-1.23	-1.09	-0.71	-1.47	-0.90	0.90
7023	TFAP4	transcription factor AP-4 [Source:HGNC Symbol;Acc:HGNC:11745]	-1.38	-1.01	-1.13	-0.66	-1.20	-0.90	0.90
8338	HIST2H2AC	histone cluster 2 H2A family member c [Source:HGNC Symbol;Acc:H	-1.34	-0.65	-1.64	-0.15	-0.99	-0.89	0.89
9319	TRIP13	thyroid hormone receptor interactor 13 [Source:HGNC Symbol;Acc	-1.59	-0.53	-1.22	-0.56	-1.06	-0.89	0.89
4176	MCM7	minichromosome maintenance complex component 7 [Source:HGN	-0.65	-1.12	-1.15	-0.64	-0.89	-0.89	0.89
83990	BRIP1	BRCA1 interacting protein C-terminal helicase 1 [Source:HGNC Sym	-1.18	-0.59	-1.40	-0.87	-0.88	-1.14	0.88
164781	DAW1	dynein assembly factor with WD repeats 1 [Source:HGNC Symbol;A	-0.79	-0.97	-1.38	-1.00	-0.88	-1.19	0.88
54734	RAB39A	RAB39A, member RAS oncogene family [Source:HGNC Symbol;Acc:	-1.05	-0.74	-1.22	-0.54	-0.89	-0.88	0.88
79075	DSCC1	DNA replication and sister chromatid cohesion 1 [Source:HGNC Syn	-0.56	-1.20	-1.46	-1.00	-0.88	-1.23	0.88
760	CA2	carbonic anhydrase 2 [Source:HGNC Symbol;Acc:HGNC:1373]	-0.79	-0.96	-1.64	-0.74	-0.88	-1.19	0.88
23649	POLA2	DNA polymerase alpha 2, accessory subunit [Source:HGNC Symbol;	-0.61	-1.17	-1.21	-0.54	-0.89	-0.88	0.88
5985	RFC5	replication factor C subunit 5 [Source:HGNC Symbol;Acc:HGNC:997	-0.83	-0.93	-1.25	-0.68	-0.88	-0.96	0.88
7298	TYMS	thymidylate synthetase [Source:HGNC Symbol;Acc:HGNC:12441]	-1.13	-0.62	-1.02	-0.72	-0.88	-0.87	0.87
4173	MCM4	minichromosome maintenance complex component 4 [Source:HGN	-0.89	-0.95	-1.14	-0.60	-0.92	-0.87	0.87
129293	TRABD2A	TraB domain containing 2A [Source:HGNC Symbol;Acc:HGNC:2701	-1.38	-0.65	-1.27	-0.47	-1.01	-0.87	0.87
22976	PAXIP1	PAX interacting protein 1 [Source:HGNC Symbol;Acc:HGNC:8624]	-1.57	-0.65	-1.02	-0.69	-1.11	-0.86	0.86
84798	C19orf48	chromosome 19 open reading frame 48 [Source:HGNC Symbol;Acc:	-0.93	-0.94	-1.24	-0.47	-0.94	-0.86	0.86
57621	ZBTB2	zinc finger and BTB domain containing 2 [Source:HGNC Symbol;Acc	-1.35	-0.47	-1.13	-0.58	-0.91	-0.85	0.85
10714	POLD3	DNA polymerase delta 3, accessory subunit [Source:HGNC Symbol;	-0.97	-0.73	-0.94	-0.87	-0.85	-0.90	0.85

4174	MCM5	minichromosome maintenance complex component 5 [Source:HGN	-0.98	-0.72	-1.41	-0.71	-0.85	-1.06	**0.85**
2175	FANCA	Fanconi anemia complementation group A [Source:HGNC Symbol;A	-1.08	-0.62	-1.42	-0.60	-0.85	-1.01	**0.85**
23007	PLCH1	phospholipase C eta 1 [Source:HGNC Symbol;Acc:HGNC:29185]	-1.19	-0.55	-0.91	-0.78	-0.87	-0.85	**0.85**
83903	HASPIN	histone H3 associated protein kinase [Source:HGNC Symbol;Acc:HG	-1.09	-0.60	-1.38	-0.52	-0.85	-0.95	**0.85**
400916	CHCHD10	coiled-coil-helix-coiled-coil-helix domain containing 10 [Source:HGN	-1.10	-0.59	-1.47	-0.76	-0.84	-1.12	**0.84**
124222	PAQR4	progestin and adipoQ receptor family member 4 [Source:HGNC Syr	-1.94	-0.60	-0.89	-0.79	-1.27	-0.84	**0.84**
64946	CENPH	centromere protein H [Source:HGNC Symbol;Acc:HGNC:17268]	-0.70	-0.97	-1.31	-0.60	-0.84	-0.95	**0.84**
63901	FAM111A	family with sequence similarity 111 member A [Source:HGNC Symb	-1.00	-0.65	-1.12	-0.63	-0.83	-0.88	**0.83**
203068	TUBB	tubulin beta class I [Source:HGNC Symbol;Acc:HGNC:20778]	-1.81	-0.07	-1.29	-0.37	-0.94	-0.83	**0.83**
4796	TONSL	tonsoku like, DNA repair protein [Source:HGNC Symbol;Acc:HGNC:	-0.88	-0.77	-1.33	-0.79	-0.83	-1.06	**0.83**
55215	FANCI	Fanconi anemia complementation group I [Source:HGNC Symbol;A	-0.87	-0.75	-1.17	-0.61	-0.81	-0.89	**0.81**
91057	CCDC34	coiled-coil domain containing 34 [Source:HGNC Symbol;Acc:HGNC:	-1.00	-0.96	-0.82	-0.81	-0.98	-0.81	**0.81**
84930	MASTL	microtubule associated serine/threonine kinase like [Source:HGNC	-0.92	-0.70	-1.56	-0.81	-0.81	-1.18	**0.81**
146956	EME1	essential meiotic structure-specific endonuclease 1 [Source:HGNC S	-0.79	-0.83	-1.31	-0.76	-0.81	-1.03	**0.81**
113115	MTFR2	mitochondrial fission regulator 2 [Source:HGNC Symbol;Acc:HGNC:	-0.94	-0.67	-1.44	-0.85	-0.81	-1.15	**0.81**
84722	PSRC1	proline and serine rich coiled-coil 1 [Source:HGNC Symbol;Acc:HGN	-1.37	-0.24	-1.75	-0.59	-0.80	-1.17	**0.80**
4172	MCM3	minichromosome maintenance complex component 3 [Source:HGN	-0.87	-0.74	-1.29	-0.68	-0.80	-0.99	**0.80**
814	CAMK4	calcium/calmodulin dependent protein kinase IV [Source:HGNC Syr	-1.76	-0.58	-0.94	-0.67	-1.17	-0.80	**0.80**
7272	TTK	TTK protein kinase [Source:HGNC Symbol;Acc:HGNC:12401]	-1.35	-0.25	-1.27	-0.66	-0.80	-0.96	**0.80**
1852	DUSP9	dual specificity phosphatase 9 [Source:HGNC Symbol;Acc:HGNC:30	-1.23	-0.37	-0.76	-0.86	-0.80	-0.81	**0.80**
9833	MELK	maternal embryonic leucine zipper kinase [Source:HGNC Symbol;A	-0.89	-0.71	-2.17	-0.60	-0.80	-1.39	**0.80**
1468	SLC25A10	solute carrier family 25 member 10 [Source:HGNC Symbol;Acc:HGN	-0.86	-0.74	-1.14	-0.55	-0.80	-0.85	**0.80**
79827	CLMP	CXADR like membrane protein [Source:HGNC Symbol;Acc:HGNC:24	-1.29	-0.30	-1.22	-0.43	-0.80	-0.83	**0.80**
2842	GPR19	G protein-coupled receptor 19 [Source:HGNC Symbol;Acc:HGNC:44	-1.07	-0.52	-1.33	-0.94	-0.79	-1.14	**0.79**
5013	OTX1	orthodenticle homeobox 1 [Source:HGNC Symbol;Acc:HGNC:8521]	-0.84	-0.74	-0.87	-0.71	-0.79	-0.79	**0.79**
26872	STEAP1	STEAP family member 1 [Source:HGNC Symbol;Acc:HGNC:11378]	-1.37	-0.21	-1.40	-0.22	-0.79	-0.81	**0.79**
147841	SPC24	SPC24, NDC80 kinetochore complex component [Source:HGNC Syr	-1.17	-0.41	-1.85	-0.61	-0.79	-1.23	**0.79**
26271	FBXO5	F-box protein 5 [Source:HGNC Symbol;Acc:HGNC:13584]	-1.04	-0.52	-1.67	-0.82	-0.78	-1.25	**0.78**
83461	CDCA3	cell division cycle associated 3 [Source:HGNC Symbol;Acc:HGNC:14	-1.20	-0.37	-1.57	-0.44	-0.78	-1.01	**0.78**
5422	POLA1	DNA polymerase alpha 1, catalytic subunit [Source:HGNC Symbol;A	-0.94	-1.20	-0.88	-0.68	-1.07	-0.78	**0.78**
9768	PCLAF	PCNA clamp associated factor [Source:HGNC Symbol;Acc:HGNC:28	-1.21	-0.63	-1.06	-0.49	-0.92	-0.77	**0.77**
8914	TIMELESS	timeless circadian regulator [Source:HGNC Symbol;Acc:HGNC:1181	-0.20	-1.35	-0.89	-0.68	-0.77	-0.78	**0.77**
3619	INCENP	inner centromere protein [Source:HGNC Symbol;Acc:HGNC:6058]	-0.97	-0.58	-1.27	-0.85	-0.77	-1.06	**0.77**
3978	LIG1	DNA ligase 1 [Source:HGNC Symbol;Acc:HGNC:6598]	-0.78	-0.76	-1.06	-0.82	-0.77	-0.94	**0.77**
8479	HIRIP3	HIRA interacting protein 3 [Source:HGNC Symbol;Acc:HGNC:4917]	-1.03	-0.51	-0.93	-0.61	-0.77	-0.77	**0.77**
5932	RBBP8	RB binding protein 8, endonuclease [Source:HGNC Symbol;Acc:HGN	-0.51	-1.03	-0.92	-0.67	-0.77	-0.80	**0.77**
701	BUB1B	BUB1 mitotic checkpoint serine/threonine kinase B [Source:HGNC S	-0.93	-0.59	-1.62	-0.53	-0.76	-1.08	**0.76**
6240	RRM1	ribonucleotide reductase catalytic subunit M1 [Source:HGNC Symb	-1.05	-0.77	-1.05	-0.48	-0.91	-0.76	**0.76**
29941	PKN3	protein kinase N3 [Source:HGNC Symbol;Acc:HGNC:17999]	-1.06	-0.47	-1.11	-0.50	-0.76	-0.80	**0.76**
81931	ZNF93	zinc finger protein 93 [Source:HGNC Symbol;Acc:HGNC:13169]	-0.83	-0.70	-1.01	-0.99	-0.76	-1.00	**0.76**
220108	FAM124A	family with sequence similarity 124 member A [Source:HGNC Symb	-0.89	-0.65	-0.89	-0.63	-0.77	-0.76	**0.76**
7161	TP73	tumor protein p73 [Source:HGNC Symbol;Acc:HGNC:12003]	-1.04	-0.48	-0.97	-0.96	-0.76	-0.97	**0.76**
54821	ERCC6L	ERCC excision repair 6 like, spindle assembly checkpoint helicase [S	-1.04	-0.48	-2.01	-0.63	-0.76	-1.32	**0.76**
23082	PPRC1	peroxisome proliferator-activated receptor gamma, coactivator-rel	-0.88	-0.64	-0.94	-0.56	-0.76	-0.75	**0.75**
23306	NEMP1	nuclear envelope integral membrane protein 1 [Source:HGNC Symb	-0.58	-0.92	-1.96	-0.60	-0.75	-1.28	**0.75**
145773	FAM81A	family with sequence similarity 81 member A [Source:HGNC Symbo	-1.09	-0.81	-0.82	-0.68	-0.95	-0.75	**0.75**
10615	SPAG5	sperm associated antigen 5 [Source:HGNC Symbol;Acc:HGNC:1345	-1.22	-0.28	-1.46	-0.48	-0.75	-0.97	**0.75**
8458	TTF2	transcription termination factor 2 [Source:HGNC Symbol;Acc:HGNC	-0.51	-0.98	-0.90	-0.92	-0.75	-0.91	**0.75**
5347	PLK1	polo like kinase 1 [Source:HGNC Symbol;Acc:HGNC:9077]	-0.57	-0.92	-1.56	-0.57	-0.74	-1.06	**0.74**
5036	PA2G4	proliferation-associated 2G4 [Source:HGNC Symbol;Acc:HGNC:855	-0.86	-1.23	-0.84	-0.65	-1.05	-0.74	**0.74**
84811	BUD13	BUD13 homolog [Source:HGNC Symbol;Acc:HGNC:28199]	-0.93	-0.56	-0.89	-0.76	-0.74	-0.82	**0.74**
64858	DCLRE1B	DNA cross-link repair 1B [Source:HGNC Symbol;Acc:HGNC:17641]	-1.14	-0.56	-0.80	-0.67	-0.85	-0.74	**0.74**
2583	B4GALNT1	beta-1,4-N-acetyl-galactosaminyltransferase 1 [Source:HGNC Symb	-1.34	-0.13	-2.31	-0.29	-0.73	-1.30	**0.73**
596	BCL2	BCL2, apoptosis regulator [Source:HGNC Symbol;Acc:HGNC:990]	-1.30	-0.72	-0.84	-0.62	-1.01	-0.73	**0.73**
5983	RFC3	replication factor C subunit 3 [Source:HGNC Symbol;Acc:HGNC:997	-0.72	-0.75	-1.30	-0.81	-0.73	-1.06	**0.73**
23046	KIF21B	kinesin family member 21B [Source:HGNC Symbol;Acc:HGNC:2944	-0.83	-0.71	-0.41	-1.05	-0.77	-0.73	**0.73**
11130	ZWINT	ZW10 interacting kinetochore protein [Source:HGNC Symbol;Acc:H	-0.51	-0.95	-2.02	-0.65	-0.73	-1.33	**0.73**
11100	HNRNPUL1	heterogeneous nuclear ribonucleoprotein U like 1 [Source:HGNC Sy	-1.99	-0.30	-0.92	-0.54	-1.15	-0.73	**0.73**
375033	PEAR1	platelet endothelial aggregation receptor 1 [Source:HGNC Symbol;	-1.57	-0.22	-1.24	-0.21	-0.89	-0.73	**0.73**
79723	SUV39H2	suppressor of variegation 3-9 homolog 2 [Source:HGNC Symbol;Acc	-0.60	-0.85	-1.01	-0.56	-0.72	-0.79	**0.72**
7112	TMPO	thymopoietin [Source:HGNC Symbol;Acc:HGNC:11875]	-0.94	-0.50	-1.55	-0.82	-0.72	-1.19	**0.72**
26528	DAZAP1	DAZ associated protein 1 [Source:HGNC Symbol;Acc:HGNC:2683]	-1.50	-0.53	-0.94	-0.51	-1.01	-0.72	**0.72**
672	BRCA1	BRCA1, DNA repair associated [Source:HGNC Symbol;Acc:HGNC:11	-0.27	-1.18	-1.00	-0.70	-0.72	-0.85	**0.72**
79832	QSER1	glutamine and serine rich 1 [Source:HGNC Symbol;Acc:HGNC:2615	-1.19	-0.26	-1.39	-0.43	-0.72	-0.91	**0.72**
79682	CENPU	centromere protein U [Source:HGNC Symbol;Acc:HGNC:21348]	-0.78	-0.66	-1.47	-1.12	-0.72	-1.29	**0.72**
6615	SNAI1	snail family transcriptional repressor 1 [Source:HGNC Symbol;Acc:H	-1.17	-0.40	-1.01	-0.42	-0.78	-0.72	**0.72**
55646	LYAR	Ly1 antibody reactive [Source:HGNC Symbol;Acc:HGNC:26021]	-2.16	-0.39	-0.94	-0.49	-1.27	-0.72	**0.72**
51176	LEF1	lymphoid enhancer binding factor 1 [Source:HGNC Symbol;Acc:HGN	-1.49	-1.83	-0.69	-0.74	-1.66	-0.71	**0.71**
9495	AKAP5	A-kinase anchoring protein 5 [Source:HGNC Symbol;Acc:HGNC:375	-1.03	-0.40	-0.85	-1.11	-0.71	-0.98	**0.71**
83695	RHNO1	RAD9-HUS1-RAD1 interacting nuclear orphan 1 [Source:HGNC Sym	-1.13	-0.31	-0.95	-0.48	-0.72	-0.71	**0.71**
9937	DCLRE1A	DNA cross-link repair 1A [Source:HGNC Symbol;Acc:HGNC:17660]	-0.89	-0.63	-0.89	-0.53	-0.76	-0.71	**0.71**
152573	SHISA3	shisa family member 3 [Source:HGNC Symbol;Acc:HGNC:25159]	-2.32	-0.69	-1.14	-0.28	-1.50	-0.71	**0.71**
22837	COBLL1	cordon-bleu WH2 repeat protein like 1 [Source:HGNC Symbol;Acc:H	-0.68	-0.84	-0.48	-0.94	-0.76	-0.71	**0.71**
678	ZFP36L2	ZFP36 ring finger protein like 2 [Source:HGNC Symbol;Acc:HGNC:11	-1.32	-0.09	-1.52	-0.48	-0.71	-1.00	**0.71**
140707	BRI3BP	BRI3 binding protein [Source:HGNC Symbol;Acc:HGNC:14251]	-0.78	-1.42	-0.48	-0.93	-1.10	-0.71	**0.71**
401505	TOMM5	translocase of outer mitochondrial membrane 5 [Source:HGNC Syr	-1.46	-0.97	-0.86	-0.56	-1.22	-0.71	**0.71**
54510	PCDH18	protocadherin 18 [Source:HGNC Symbol;Acc:HGNC:14268]	-2.21	-1.36	-1.06	-0.36	-1.79	-0.71	**0.71**
130340	AP1S3	adaptor related protein complex 1 sigma 3 subunit [Source:HGNC S	-0.89	-0.53	-0.72	-0.69	-0.71	-0.70	**0.70**
90381	TICRR	TOPBP1 interacting checkpoint and replication regulator [Source:H	-0.68	-0.72	-1.26	-0.56	-0.70	-0.91	**0.70**
3609	ILF3	interleukin enhancer binding factor 3 [Source:HGNC Symbol;Acc:H	-0.56	-0.85	-1.02	-0.38	-0.70	-0.70	**0.70**
80010	RMI1	RecQ mediated genome instability 1 [Source:HGNC Symbol;Acc:HG	-0.79	-0.62	-0.85	-0.70	-0.70	-0.77	**0.70**
83541	FAM110A	family with sequence similarity 110 member A [Source:HGNC Symb	-0.98	-0.42	-1.24	-0.73	-0.70	-0.98	**0.70**
253714	MMS22L	MMS22 like, DNA repair protein [Source:HGNC Symbol;Acc:HGNC:	-0.69	-0.70	-1.35	-0.56	-0.70	-0.95	**0.70**
151230	KLHL23	kelch like family member 23 [Source:HGNC Symbol;Acc:HGNC:275	-0.64	-1.00	-0.85	-0.54	-0.82	-0.70	**0.70**
84515	MCM8	minichromosome maintenance 8 homologous recombination repai	-0.44	-0.96	-1.00	-0.66	-0.70	-0.83	**0.70**
348487	FAM131C	family with sequence similarity 131 member C [Source:HGNC Symb	-1.18	-0.21	-1.20	-0.22	-0.70	-0.71	**0.70**
4175	MCM6	minichromosome maintenance complex component 6 [Source:HGN	-0.79	-0.92	-0.68	-0.71	-0.86	-0.70	**0.70**
11217	AKAP2	A-kinase anchoring protein 2 [Source:HGNC Symbol;Acc:HGNC:372	-1.82	-0.81	-0.63	-0.76	-1.32	-0.70	**0.70**
119	ADD2	adducin 2 [Source:HGNC Symbol;Acc:HGNC:244]	-1.38	-0.39	-0.94	-0.45	-0.88	-0.69	**0.69**
199953	TMEM201	transmembrane protein 201 [Source:HGNC Symbol;Acc:HGNC:337	-0.68	-0.89	-0.77	-0.62	-0.79	-0.69	**0.69**
23329	TBC1D30	TBC1 domain family member 30 [Source:HGNC Symbol;Acc:HGNC:	-1.17	-1.48	-0.63	-0.75	-1.32	-0.69	**0.69**

26577	PCOLCE2	procollagen C-endopeptidase enhancer 2 [Source:HGNC Symbol;Ac	-1.87	-0.34	-0.78	-0.59	-1.10	-0.69	**0.69**
5757	PTMA	prothymosin, alpha [Source:HGNC Symbol;Acc:HGNC:9623]	-1.43	-0.72	-0.77	-0.61	-1.07	-0.69	**0.69**
5425	POLD2	DNA polymerase delta 2, accessory subunit [Source:HGNC Symbol;/	-1.12	-0.66	-1.03	-0.34	-0.89	-0.69	**0.69**
57544	TXNDC16	thioredoxin domain containing 16 [Source:HGNC Symbol;Acc:HGNC	-0.77	-0.60	-1.13	-0.34	-0.69	-0.73	**0.69**
7913	DEK	DEK proto-oncogene [Source:HGNC Symbol;Acc:HGNC:2768]	-0.94	-0.43	-1.51	-0.54	-0.69	-1.02	**0.69**
3182	HNRNPAB	heterogeneous nuclear ribonucleoprotein A/B [Source:HGNC Symb	-0.88	-0.50	-0.98	-0.55	-0.69	-0.77	**0.69**
81611	ANP32E	acidic nuclear phosphoprotein 32 family member E [Source:HGNC S	-1.06	-0.31	-1.64	-0.52	-0.69	-1.08	**0.69**
3184	HNRNPD	heterogeneous nuclear ribonucleoprotein D [Source:HGNC Symbol;	-1.09	-0.44	-0.78	-0.59	-0.76	-0.69	**0.69**
1104	RCC1	regulator of chromosome condensation 1 [Source:HGNC Symbol;Ac	-2.51	-0.72	-0.93	-0.44	-1.62	-0.68	**0.68**
7351	UCP2	uncoupling protein 2 [Source:HGNC Symbol;Acc:HGNC:12518]	-0.58	-0.79	-1.01	-0.48	-0.68	-0.75	**0.68**
84057	MND1	meiotic nuclear divisions 1 [Source:HGNC Symbol;Acc:HGNC:24839	-0.77	-0.60	-1.31	-0.80	-0.68	-1.05	**0.68**
54962	TIPIN	TIMELESS interacting protein [Source:HGNC Symbol;Acc:HGNC:307	-0.55	-0.81	-0.83	-0.53	-0.68	-0.68	**0.68**
7027	TFDP1	transcription factor Dp-1 [Source:HGNC Symbol;Acc:HGNC:11749]	-0.78	-0.67	-0.96	-0.41	-0.73	-0.68	**0.68**
90488	TMEM263	transmembrane protein 263 [Source:HGNC Symbol;Acc:HGNC:2828	-0.67	-0.69	-1.20	-0.49	-0.68	-0.84	**0.68**
1663	DDX11	DEAD/H-box helicase 11 [Source:HGNC Symbol;Acc:HGNC:2736]	-0.60	-0.77	-0.79	-0.57	-0.69	-0.68	**0.68**
51540	SCLY	selenocysteine lyase [Source:HGNC Symbol;Acc:HGNC:18161]	-0.66	-1.11	-0.82	-0.53	-0.88	-0.68	**0.68**
79172	CENPO	centromere protein O [Source:HGNC Symbol;Acc:HGNC:28152]	-0.83	-0.52	-0.98	-0.49	-0.68	-0.73	**0.68**
22974	TPX2	TPX2, microtubule nucleation factor [Source:HGNC Symbol;Acc:HG	-1.00	-0.36	-1.38	-0.60	-0.68	-0.99	**0.68**
64782	AEN	apoptosis enhancing nuclease [Source:HGNC Symbol;Acc:HGNC:25	-0.86	-0.71	-0.79	-0.56	-0.79	-0.68	**0.68**
133522	PPARGC1B	PPARG coactivator 1 beta [Source:HGNC Symbol;Acc:HGNC:30022]	-0.53	-1.25	-0.82	-0.54	-0.89	-0.68	**0.68**
84823	LMNB2	lamin B2 [Source:HGNC Symbol;Acc:HGNC:6638]	-0.78	-0.57	-0.92	-0.55	-0.68	-0.73	**0.68**
27166	PRELID1	PRELI domain containing 1 [Source:HGNC Symbol;Acc:HGNC:30255	-1.08	-0.27	-1.35	-0.22	-0.68	-0.79	**0.68**
201161	CENPV	centromere protein V [Source:HGNC Symbol;Acc:HGNC:29920]	-0.94	-0.61	-0.90	-0.44	-0.78	-0.67	**0.67**
129401	NUP35	nucleoporin 35 [Source:HGNC Symbol;Acc:HGNC:29797]	-1.68	-1.91	-0.83	-0.51	-1.80	-0.67	**0.67**
29028	ATAD2	ATPase family, AAA domain containing 2 [Source:HGNC Symbol;Acc	-0.72	-0.61	-1.02	-0.76	-0.67	-0.89	**0.67**
200916	RPL22L1	ribosomal protein L22 like 1 [Source:HGNC Symbol;Acc:HGNC:2761	-1.03	-0.60	-1.01	-0.32	-0.82	-0.66	**0.66**
91752	ZNF804A	zinc finger protein 804A [Source:HGNC Symbol;Acc:HGNC:21711]	-0.84	-0.96	-0.56	-0.77	-0.90	-0.66	**0.66**
79000	AUNIP	aurora kinase A and ninein interacting protein [Source:HGNC Symb	-0.36	-0.96	-1.47	-0.69	-0.66	-1.08	**0.66**
2774	GNAL	G protein subunit alpha L [Source:HGNC Symbol;Acc:HGNC:4388]	-1.41	-0.29	-0.81	-0.52	-0.85	-0.66	**0.66**
3589	IL11	interleukin 11 [Source:HGNC Symbol;Acc:HGNC:5966]	-1.23	-0.09	-0.76	-0.56	-0.66	-0.66	**0.66**
11168	PSIP1	PC4 and SFRS1 interacting protein 1 [Source:HGNC Symbol;Acc:HGI	-0.83	-0.49	-0.97	-0.68	-0.66	-0.83	**0.66**
3214	HOXB4	homeobox B4 [Source:HGNC Symbol;Acc:HGNC:5115]	-1.07	-0.25	-0.55	-0.78	-0.66	-0.66	**0.66**
93594	TBC1D31	TBC1 domain family member 31 [Source:HGNC Symbol;Acc:HGNC:3	-0.62	-0.69	-1.04	-0.68	-0.65	-0.86	**0.65**
10376	TUBA1B	tubulin alpha 1b [Source:HGNC Symbol;Acc:HGNC:18809]	-1.05	-0.26	-1.24	-0.34	-0.65	-0.79	**0.65**
5888	RAD51	RAD51 recombinase [Source:HGNC Symbol;Acc:HGNC:9817]	-0.98	-0.32	-1.24	-0.47	-0.65	-0.86	**0.65**
347240	KIF24	kinesin family member 24 [Source:HGNC Symbol;Acc:HGNC:19916]	-0.79	-0.51	-1.12	-0.66	-0.65	-0.89	**0.65**
91107	TRIM47	tripartite motif containing 47 [Source:HGNC Symbol;Acc:HGNC:190	-1.33	-0.13	-1.09	-0.21	-0.73	-0.65	**0.65**
56911	MAP3K7CL	MAP3K7 C-terminal like [Source:HGNC Symbol;Acc:HGNC:16457]	-1.18	-0.12	-0.70	-1.17	-0.65	-0.93	**0.65**
79915	ATAD5	ATPase family, AAA domain containing 5 [Source:HGNC Symbol;Acc	-0.55	-0.75	-1.28	-0.68	-0.65	-0.98	**0.65**
55796	MBNL3	muscleblind like splicing regulator 3 [Source:HGNC Symbol;Acc:HGI	-0.76	-0.67	-0.94	-0.37	-0.71	-0.65	**0.65**
23397	NCAPH	non-SMC condensin I complex subunit H [Source:HGNC Symbol;Acc	-0.64	-0.66	-1.83	-0.72	-0.65	-1.27	**0.65**
4833	NME4	NME/NM23 nucleoside diphosphate kinase 4 [Source:HGNC Symbo	-1.64	-0.45	-1.10	-0.21	-1.05	-0.65	**0.65**
5982	RFC2	replication factor C subunit 2 [Source:HGNC Symbol;Acc:HGNC:997	-0.65	-0.66	-1.14	-0.71	-0.65	-0.92	**0.65**
64105	CENPK	centromere protein K [Source:HGNC Symbol;Acc:HGNC:29479]	-0.76	-0.53	-1.02	-0.73	-0.65	-0.88	**0.65**
5424	POLD1	DNA polymerase delta 1, catalytic subunit [Source:HGNC Symbol;A	-0.70	-0.60	-0.85	-0.62	-0.65	-0.73	**0.65**
23178	PASK	PAS domain containing serine/threonine kinase [Source:HGNC Sym	-0.55	-0.93	-0.53	-0.76	-0.74	-0.65	**0.65**
25788	RAD54B	RAD54 homolog B (S. cerevisiae) [Source:HGNC Symbol;Acc:HGNC:	-0.47	-0.82	-1.31	-0.87	-0.65	-1.09	**0.65**
2736	GLI2	GLI family zinc finger 2 [Source:HGNC Symbol;Acc:HGNC:4318]	-1.32	-0.29	-0.77	-0.52	-0.81	-0.65	**0.65**
10189	ALYREF	Aly/REF export factor [Source:HGNC Symbol;Acc:HGNC:19071]	-1.01	-0.28	-0.83	-0.59	-0.65	-0.71	**0.65**
10606	PAICS	phosphoribosylaminoimidazole carboxylase and phosphoribosylam	-1.21	-0.78	-0.71	-0.58	-0.99	-0.64	**0.64**
27229	TUBGCP4	tubulin gamma complex associated protein 4 [Source:HGNC Symbo	-0.69	-1.01	-0.89	-0.39	-0.85	-0.64	**0.64**
10236	HNRNPR	heterogeneous nuclear ribonucleoprotein R [Source:HGNC Symbol;	-0.99	-0.30	-0.82	-0.49	-0.64	-0.65	**0.64**
6510	SLC1A5	solute carrier family 1 member 5 [Source:HGNC Symbol;Acc:HGNC:	-1.27	-0.18	-1.10	-0.18	-0.73	-0.64	**0.64**
1763	DNA2	DNA replication helicase/nuclease 2 [Source:HGNC Symbol;Acc:HGI	-0.40	-0.88	-1.21	-0.69	-0.64	-0.95	**0.64**
55723	ASF1B	anti-silencing function 1B histone chaperone [Source:HGNC Symbo	-0.75	-0.53	-1.73	-0.56	-0.64	-1.15	**0.64**
220042	DDIAS	DNA damage induced apoptosis suppressor [Source:HGNC Symbol;	-0.54	-0.73	-1.67	-0.86	-0.64	-1.26	**0.64**
122953	JDP2	Jun dimerization protein 2 [Source:HGNC Symbol;Acc:HGNC:17546	-0.82	-0.46	-0.79	-0.95	-0.64	-0.87	**0.64**
85444	LRRCC1	leucine rich repeat and coiled-coil centrosomal protein 1 [Source:H	-0.59	-0.68	-0.91	-0.71	-0.64	-0.81	**0.64**
27316	RBMX	RNA binding motif protein, X-linked [Source:HGNC Symbol;Acc:HGN	-0.90	-0.37	-1.05	-0.27	-0.64	-0.66	**0.64**
7514	XPO1	exportin 1 [Source:HGNC Symbol;Acc:HGNC:12825]	-0.50	-0.86	-0.85	-0.42	-0.68	-0.63	**0.63**
64151	NCAPG	non-SMC condensin I complex subunit G [Source:HGNC Symbol;Acc	-0.65	-0.60	-1.61	-0.60	-0.63	-1.10	**0.63**
23212	RRS1	ribosome biogenesis regulator homolog [Source:HGNC Symbol;Acc	-0.62	-0.98	-0.62	-0.63	-0.80	-0.63	**0.63**
51512	GTSE1	G2 and S-phase expressed 1 [Source:HGNC Symbol;Acc:HGNC:1369	-0.79	-0.46	-1.55	-0.54	-0.62	-1.05	**0.62**
23594	ORC6	origin recognition complex subunit 6 [Source:HGNC Symbol;Acc:HG	-0.57	-0.68	-1.31	-0.81	-0.62	-1.06	**0.62**
8624	PSMG1	proteasome assembly chaperone 1 [Source:HGNC Symbol;Acc:HGN	-0.56	-0.69	-0.81	-0.67	-0.62	-0.74	**0.62**
81563	C1orf21	chromosome 1 open reading frame 21 [Source:HGNC Symbol;Acc:H	-1.24	-0.33	-0.51	-0.73	-0.79	-0.62	**0.62**
874	CBR3	carbonyl reductase 3 [Source:HGNC Symbol;Acc:HGNC:1549]	-1.13	-0.11	-0.92	-0.52	-0.62	-0.72	**0.62**
375444	C5orf34	chromosome 5 open reading frame 34 [Source:HGNC Symbol;Acc:H	-0.53	-0.71	-1.15	-0.76	-0.62	-0.95	**0.62**
9837	GINS1	GINS complex subunit 1 [Source:HGNC Symbol;Acc:HGNC:28980]	-0.62	-0.62	-1.67	-0.67	-0.62	-1.17	**0.62**
1017	CDK2	cyclin dependent kinase 2 [Source:HGNC Symbol;Acc:HGNC:1771]	-0.94	-0.30	-0.99	-0.61	-0.62	-0.80	**0.62**
54492	NEURL1B	neuralized E3 ubiquitin protein ligase 1B [Source:HGNC Symbol;Acc	-1.06	-0.18	-2.25	-0.83	-0.62	-1.54	**0.62**
1676	DFFA	DNA fragmentation factor subunit alpha [Source:HGNC Symbol;Acc	-1.00	-0.64	-0.72	-0.52	-0.82	-0.62	**0.62**
79980	DSN1	DSN1 homolog, MIS12 kinetochore complex component [Source:H	-0.58	-0.65	-1.10	-0.61	-0.62	-0.86	**0.62**
10112	KIF20A	kinesin family member 20A [Source:HGNC Symbol;Acc:HGNC:9787]	-1.06	-0.17	-1.58	-0.50	-0.62	-1.04	**0.62**
NA	RMI2	RecQ mediated genome instability 2 [Source:HGNC Symbol;Acc:HG	-1.58	-0.39	-0.75	-0.49	-0.99	-0.62	**0.62**
4522	MTHFD1	methylenetetrahydrofolate dehydrogenase, cyclohydrolase and for	-1.09	-0.68	-0.78	-0.46	-0.89	-0.62	**0.62**
146909	KIF18B	kinesin family member 18B [Source:HGNC Symbol;Acc:HGNC:2710	-0.88	-0.35	-1.94	-0.69	-0.62	-1.32	**0.62**
23354	HAUS5	HAUS augmin like complex subunit 5 [Source:HGNC Symbol;Acc:HG	-0.65	-0.58	-0.73	-0.51	-0.62	-0.62	**0.62**
3159	HMGA1	high mobility group AT-hook 1 [Source:HGNC Symbol;Acc:HGNC:50	-1.83	-1.03	-1.22	-0.01	-1.43	-0.62	**0.62**
10721	POLQ	DNA polymerase theta [Source:HGNC Symbol;Acc:HGNC:9186]	-0.41	-0.83	-1.46	-0.58	-0.62	-1.02	**0.62**
4218	RAB8A	RAB8A, member RAS oncogene family [Source:HGNC Symbol;Acc:H	-2.01	-0.14	-0.82	-0.41	-1.08	-0.62	**0.62**
9532	BAG2	BCL2 associated athanogene 2 [Source:HGNC Symbol;Acc:HGNC:93	-1.66	-0.57	-0.71	-0.52	-1.12	-0.61	**0.61**
6382	SDC1	syndecan 1 [Source:HGNC Symbol;Acc:HGNC:10658]	-1.23	0.00	-0.86	-1.01	-0.61	-0.93	**0.61**
6632	SNRPD1	small nuclear ribonucleoprotein D1 polypeptide [Source:HGNC Sym	-0.70	-0.64	-0.67	-0.56	-0.67	-0.61	**0.61**
6059	ABCE1	ATP binding cassette subfamily E member 1 [Source:HGNC Symbol;	-0.60	-0.67	-0.95	-0.27	-0.63	-0.61	**0.61**
29980	DONSON	downstream neighbor of SON [Source:HGNC Symbol;Acc:HGNC:29	-0.62	-0.60	-1.12	-0.49	-0.61	-0.80	**0.61**
11004	KIF2C	kinesin family member 2C [Source:HGNC Symbol;Acc:HGNC:6393]	-0.62	-0.59	-1.61	-0.54	-0.61	-1.07	**0.61**
10635	RAD51AP1	RAD51 associated protein 1 [Source:HGNC Symbol;Acc:HGNC:1695	-0.51	-0.71	-1.86	-0.79	-0.61	-1.32	**0.61**
2091	FBL	fibrillarin [Source:HGNC Symbol;Acc:HGNC:3599]	-1.02	-0.68	-0.74	-0.47	-0.85	-0.61	**0.61**
54892	NCAPG2	non-SMC condensin II complex subunit G2 [Source:HGNC Symbol;A	-0.96	-0.25	-1.15	-0.46	-0.61	-0.81	**0.61**
10056	FARSB	phenylalanyl-tRNA synthetase beta subunit [Source:HGNC Symbol;/	-0.34	-0.87	-1.03	-0.36	-0.61	-0.69	**0.61**

1736	DKC1	dyskerin pseudouridine synthase 1 [Source:HGNC Symbol;Acc:HGN	-0.64	-0.56	-1.10	-0.29	-0.60	-0.69	**0.60**
55809	TRERF1	transcriptional regulating factor 1 [Source:HGNC Symbol;Acc:HGNC	-1.44	-0.01	-1.17	-0.03	-0.72	-0.60	**0.60**
8645	KCNK5	potassium two pore domain channel subfamily K member 5 [Source	-0.90	-0.31	-0.88	-0.88	-0.60	-0.88	**0.60**
5831	PYCR1	pyrroline-5-carboxylate reductase 1 [Source:HGNC Symbol;Acc:HGI	-1.06	-0.15	-0.93	-0.27	-0.61	-0.60	**0.60**
57513	CASKIN2	CASK interacting protein 2 [Source:HGNC Symbol;Acc:HGNC:18200	-0.84	-0.42	-0.80	-0.40	-0.63	-0.60	**0.60**
2118	ETV4	ETS variant 4 [Source:HGNC Symbol;Acc:HGNC:3493]	-0.89	-0.31	-0.93	-0.31	-0.60	-0.62	**0.60**
50486	G0S2	G0/G1 switch 2 [Source:HGNC Symbol;Acc:HGNC:30229]	-3.02	-0.23	-1.14	-0.06	-1.62	-0.60	**0.60**
55167	MSL2	MSL complex subunit 2 [Source:HGNC Symbol;Acc:HGNC:25544]	-0.87	-0.40	-0.84	-0.36	-0.64	-0.60	**0.60**
2189	FANCG	Fanconi anemia complementation group G [Source:HGNC Symbol;A	-0.56	-0.64	-1.37	-0.60	-0.60	-0.98	**0.60**
84131	CEP78	centrosomal protein 78 [Source:HGNC Symbol;Acc:HGNC:25740]	-0.67	-0.62	-0.66	-0.54	-0.65	-0.60	**0.60**
26150	RIBC2	RIB43A domain with coiled-coils 2 [Source:HGNC Symbol;Acc:HGNC	-0.58	-0.70	-0.97	-0.23	-0.64	-0.60	**0.60**
389831	AC011043.1	Homo sapiens uncharacterized LOC389831 (LOC389831), transcript	-0.68	-0.55	-0.82	-0.38	-0.61	-0.60	**0.60**
6732	SRPK1	SRSF protein kinase 1 [Source:HGNC Symbol;Acc:HGNC:11305]	-1.01	-0.56	-0.97	-0.22	-0.79	-0.60	**0.60**
10523	CHERP	calcium homeostasis endoplasmic reticulum protein [Source:HGNC	-0.31	-0.94	-0.48	-0.71	-0.62	-0.60	**0.60**
79077	DCTPP1	dCTP pyrophosphatase 1 [Source:HGNC Symbol;Acc:HGNC:28777]	-0.73	-0.63	-0.66	-0.53	-0.68	-0.59	**0.59**
5990	RFX2	regulatory factor X2 [Source:HGNC Symbol;Acc:HGNC:9983]	-1.11	-0.39	-0.76	-0.42	-0.75	-0.59	**0.59**
9590	AKAP12	A-kinase anchoring protein 12 [Source:HGNC Symbol;Acc:HGNC:37	-0.97	-0.21	-1.33	-0.11	-0.59	-0.72	**0.59**
55835	CENPJ	centromere protein J [Source:HGNC Symbol;Acc:HGNC:17272]	-0.50	-0.68	-0.79	-0.53	-0.59	-0.66	**0.59**
79080	CCDC86	coiled-coil domain containing 86 [Source:HGNC Symbol;Acc:HGNC:	-1.63	-0.78	-0.62	-0.56	-1.20	-0.59	**0.59**
55010	PARPBP	PARP1 binding protein [Source:HGNC Symbol;Acc:HGNC:26074]	-0.45	-0.73	-1.06	-0.60	-0.59	-0.83	**0.59**
675	BRCA2	BRCA2, DNA repair associated [Source:HGNC Symbol;Acc:HGNC:11	-0.29	-0.89	-1.17	-0.81	-0.59	-0.99	**0.59**
55034	MOCOS	molybdenum cofactor sulfurase [Source:HGNC Symbol;Acc:HGNC:1	-1.10	-0.53	-0.77	-0.41	-0.81	-0.59	**0.59**
286826	LIN9	lin-9 DREAM MuvB core complex component [Source:HGNC Symbo	-0.58	-0.59	-1.08	-0.71	-0.59	-0.90	**0.59**
126789	PUSL1	pseudouridylate synthase-like 1 [Source:HGNC Symbol;Acc:HGNC:2	-0.99	-0.19	-1.00	-0.44	-0.59	-0.72	**0.59**
9401	RECQL4	RecQ like helicase 4 [Source:HGNC Symbol;Acc:HGNC:9949]	-0.83	-0.34	-1.29	-0.61	-0.58	-0.95	**0.58**
10432	RBM14	RNA binding motif protein 14 [Source:HGNC Symbol;Acc:HGNC:142	-0.74	-0.44	-0.70	-0.46	-0.59	-0.58	**0.58**
57452	GALNT16	polypeptide N-acetylgalactosaminyltransferase 16 [Source:HGNC S	-1.73	-0.24	-0.64	-0.52	-0.98	-0.58	**0.58**
10051	SMC4	structural maintenance of chromosomes 4 [Source:HGNC Symbol;A	-0.84	-0.33	-1.33	-0.62	-0.58	-0.97	**0.58**
4678	NASP	nuclear autoantigenic sperm protein [Source:HGNC Symbol;Acc:HG	-0.48	-0.69	-0.74	-0.70	-0.58	-0.72	**0.58**
5321	PLA2G4A	phospholipase A2 group IVA [Source:HGNC Symbol;Acc:HGNC:9035	-0.95	-1.24	-0.73	-0.43	-1.09	-0.58	**0.58**
54205	CYCS	cytochrome c, somatic [Source:HGNC Symbol;Acc:HGNC:19986]	-0.62	-0.54	-1.06	-0.36	-0.58	-0.71	**0.58**
7283	TUBG1	tubulin gamma 1 [Source:HGNC Symbol;Acc:HGNC:12417]	-0.81	-0.35	-1.25	-0.56	-0.58	-0.91	**0.58**
10682	EBP	emopamil binding protein (sterol isomerase) [Source:HGNC Symbo	-0.94	-0.37	-0.72	-0.44	-0.65	-0.58	**0.58**
3312	HSPA8	heat shock protein family A (Hsp70) member 8 [Source:HGNC Symb	-0.75	-0.41	-0.90	-0.50	-0.58	-0.70	**0.58**
3033	HADH	hydroxyacyl-CoA dehydrogenase [Source:HGNC Symbol;Acc:HGNC:	-0.44	-0.72	-0.49	-0.97	-0.58	-0.73	**0.58**
2187	FANCB	Fanconi anemia complementation group B [Source:HGNC Symbol;A	-0.54	-0.62	-1.28	-0.58	-0.58	-0.93	**0.58**
2542	SLC37A4	solute carrier family 37 member 4 [Source:HGNC Symbol;Acc:HGNC	-0.82	-0.38	-0.53	-0.62	-0.60	-0.58	**0.58**
4691	NCL	nucleolin [Source:HGNC Symbol;Acc:HGNC:7667]	-0.64	-0.88	-0.61	-0.55	-0.76	-0.58	**0.58**
8243	SMC1A	structural maintenance of chromosomes 1A [Source:HGNC Symbol;	-0.57	-0.58	-0.75	-0.44	-0.58	-0.59	**0.58**
81610	FAM83D	family with sequence similarity 83 member D [Source:HGNC Symbo	-0.73	-0.42	-1.44	-0.53	-0.58	-0.99	**0.58**
126382	NR2C2AP	nuclear receptor 2C2 associated protein [Source:HGNC Symbol;Acc	-0.78	-0.64	-0.64	-0.52	-0.71	-0.58	**0.58**
55055	ZWILCH	zwilch kinetochore protein [Source:HGNC Symbol;Acc:HGNC:25468	-0.54	-0.74	-0.81	-0.34	-0.64	-0.58	**0.58**
2146	EZH2	enhancer of zeste 2 polycomb repressive complex 2 subunit [Source	-0.58	-0.57	-1.79	-0.59	-0.57	-1.19	**0.57**
8089	YEATS4	YEATS domain containing 4 [Source:HGNC Symbol;Acc:HGNC:24859	-0.65	-0.51	-0.56	-0.58	-0.58	-0.57	**0.57**
4085	MAD2L1	mitotic arrest deficient 2 like 1 [Source:HGNC Symbol;Acc:HGNC:67	-0.47	-0.68	-0.96	-0.62	-0.57	-0.79	**0.57**
65260	COA7	cytochrome c oxidase assembly factor 7 (putative) [Source:HGNC S	-0.50	-0.64	-0.87	-0.40	-0.57	-0.64	**0.57**
3219	HOXB9	homeobox B9 [Source:HGNC Symbol;Acc:HGNC:5120]	-0.63	-0.66	-0.83	-0.32	-0.64	-0.57	**0.57**
9735	KNTC1	kinetochore associated 1 [Source:HGNC Symbol;Acc:HGNC:17255]	-0.57	-0.58	-1.21	-0.56	-0.57	-0.89	**0.57**
51602	NOP58	NOP58 ribonucleoprotein [Source:HGNC Symbol;Acc:HGNC:29926]	-0.51	-0.64	-1.21	-0.39	-0.57	-0.80	**0.57**
494115	RBMXL1	RNA binding motif protein, X-linked like 1 [Source:HGNC Symbol;Ac	-0.87	-0.74	-0.76	-0.39	-0.80	-0.57	**0.57**
64222	TOR3A	torsin family 3 member A [Source:HGNC Symbol;Acc:HGNC:11997]	-0.62	-0.52	-0.90	-0.26	-0.57	-0.58	**0.57**
79019	CENPM	centromere protein M [Source:HGNC Symbol;Acc:HGNC:18352]	-0.79	-0.35	-1.09	-0.59	-0.57	-0.84	**0.57**
3832	KIF11	kinesin family member 11 [Source:HGNC Symbol;Acc:HGNC:6388]	-0.51	-0.64	-1.61	-0.62	-0.57	-1.11	**0.57**
55157	DARS2	aspartyl-tRNA synthetase 2, mitochondrial [Source:HGNC Symbol;A	-0.66	-0.65	-0.77	-0.37	-0.65	-0.57	**0.57**
4849	CNOT3	CCR4-NOT transcription complex subunit 3 [Source:HGNC Symbol;A	-1.28	-0.75	-0.63	-0.51	-1.02	-0.57	**0.57**
122769	LRR1	leucine rich repeat protein 1 [Source:HGNC Symbol;Acc:HGNC:1974	-0.91	-0.23	-0.76	-0.51	-0.57	-0.63	**0.57**
6749	SSRP1	structure specific recognition protein 1 [Source:HGNC Symbol;Acc:I	-1.01	-0.54	-0.72	-0.42	-0.77	-0.57	**0.57**
7480	WNT10B	Wnt family member 10B [Source:HGNC Symbol;Acc:HGNC:12775]	-1.26	-0.51	-0.79	-0.34	-0.88	-0.57	**0.57**
580	BARD1	BRCA1 associated RING domain 1 [Source:HGNC Symbol;Acc:HGNC	-0.67	-0.46	-1.14	-0.64	-0.57	-0.89	**0.57**
27341	RRP7A	ribosomal RNA processing 7 homolog A [Source:HGNC Symbol;Acc:	-0.49	-0.64	-0.71	-0.51	-0.56	-0.61	**0.56**
79180	EFHD2	EF-hand domain family member D2 [Source:HGNC Symbol;Acc:HGN	-0.96	-0.17	-1.40	-1.03	-0.56	-1.21	**0.56**
5426	POLE	DNA polymerase epsilon, catalytic subunit [Source:HGNC Symbol;A	-0.65	-0.47	-1.14	-0.65	-0.56	-0.90	**0.56**
54839	LRRC49	leucine rich repeat containing 49 [Source:HGNC Symbol;Acc:HGNC:	-0.85	-0.87	-0.68	-0.45	-0.86	-0.56	**0.56**
55700	MAP7D1	MAP7 domain containing 1 [Source:HGNC Symbol;Acc:HGNC:25514	-1.44	-0.01	-0.66	-0.47	-0.73	-0.56	**0.56**
22995	CEP152	centrosomal protein 152 [Source:HGNC Symbol;Acc:HGNC:29298]	-0.57	-0.55	-0.82	-0.57	-0.56	-0.69	**0.56**
23310	NCAPD3	non-SMC condensin II complex subunit D3 [Source:HGNC Symbol;A	-0.34	-0.78	-1.22	-0.59	-0.56	-0.90	**0.56**
9754	STARD8	StAR related lipid transfer domain containing 8 [Source:HGNC Symb	-0.64	-0.48	-0.97	-0.37	-0.56	-0.67	**0.56**
5902	RANBP1	RAN binding protein 1 [Source:HGNC Symbol;Acc:HGNC:9847]	-0.95	-0.65	-0.50	-0.61	-0.80	-0.56	**0.56**
55789	DEPDC1B	DEP domain containing 1B [Source:HGNC Symbol;Acc:HGNC:24902	-0.48	-0.64	-1.18	-0.47	-0.56	-0.82	**0.56**
113130	CDCA5	cell division cycle associated 5 [Source:HGNC Symbol;Acc:HGNC:14	-0.66	-0.46	-1.51	-0.70	-0.56	-1.11	**0.56**
3148	HMGB2	high mobility group box 2 [Source:HGNC Symbol;Acc:HGNC:5000]	-0.86	-0.25	-0.67	-0.59	-0.56	-0.63	**0.56**
57673	BEND3	BEN domain containing 3 [Source:HGNC Symbol;Acc:HGNC:23040]	-0.92	-0.65	-0.92	-0.20	-0.79	-0.56	**0.56**
219790	RTKN2	rhotekin 2 [Source:HGNC Symbol;Acc:HGNC:19364]	-0.54	-0.57	-0.78	-0.95	-0.55	-0.86	**0.55**
8187	ZNF239	zinc finger protein 239 [Source:HGNC Symbol;Acc:HGNC:13031]	-0.28	-0.83	-1.13	-0.53	-0.55	-0.83	**0.55**
55732	C1orf112	chromosome 1 open reading frame 112 [Source:HGNC Symbol;Acc:	-0.47	-0.64	-1.57	-0.45	-0.55	-1.01	**0.55**
51020	HDDC2	HD domain containing 2 [Source:HGNC Symbol;Acc:HGNC:21078]	-0.99	-0.67	-0.83	-0.28	-0.83	-0.55	**0.55**
10212	DDX39A	DExD-box helicase 39A [Source:HGNC Symbol;Acc:HGNC:17821]	-0.83	-0.27	-1.07	-0.40	-0.55	-0.73	**0.55**
57650	CIP2A	cell proliferation regulating inhibitor of protein phosphatase 2A [So	-0.69	-0.41	-1.64	-0.69	-0.55	-1.16	**0.55**
9972	NUP153	nucleoporin 153 [Source:HGNC Symbol;Acc:HGNC:8062]	-0.77	-0.50	-0.67	-0.43	-0.64	-0.55	**0.55**
8704	B4GALT2	beta-1,4-galactosyltransferase 2 [Source:HGNC Symbol;Acc:HGNC:9	-0.91	-0.19	-1.21	-0.39	-0.55	-0.80	**0.55**
9685	CLINT1	clathrin interactor 1 [Source:HGNC Symbol;Acc:HGNC:23186]	-1.09	-0.01	-0.99	-0.11	-0.55	-0.55	**0.55**
10736	SIX2	SIX homeobox 2 [Source:HGNC Symbol;Acc:HGNC:10888]	-2.02	-0.69	-0.56	-0.54	-1.36	-0.55	**0.55**
27085	MTBP	MDM2 binding protein [Source:HGNC Symbol;Acc:HGNC:7417]	-0.26	-0.83	-0.75	-0.59	-0.55	-0.67	**0.55**
55143	CDCA8	cell division cycle associated 8 [Source:HGNC Symbol;Acc:HGNC:14	-0.68	-0.42	-1.49	-0.42	-0.55	-0.95	**0.55**
1719	DHFR	dihydrofolate reductase [Source:HGNC Symbol;Acc:HGNC:2861]	-0.57	-0.56	-0.61	-0.49	-0.57	-0.55	**0.55**
643853	TMPPE	transmembrane protein with metallophosphoesterase domain [Sou	-1.30	-0.26	-0.90	-0.20	-0.78	-0.55	**0.55**
23616	SH3BP1	SH3 domain binding protein 1 [Source:HGNC Symbol;Acc:HGNC:10	-1.14	-0.16	-0.61	-0.48	-0.65	-0.55	**0.55**
5496	PPM1G	protein phosphatase, Mg2+/Mn2+ dependent 1G [Source:HGNC Sy	-0.57	-0.71	-0.57	-0.52	-0.64	-0.54	**0.54**
201725	C4orf46	chromosome 4 open reading frame 46 [Source:HGNC Symbol;Acc:H	-0.58	-0.50	-1.43	-0.82	-0.54	-1.13	**0.54**
9928	KIF14	kinesin family member 14 [Source:HGNC Symbol;Acc:HGNC:19181]	-0.81	-0.28	-1.31	-0.54	-0.54	-0.93	**0.54**
55589	BMP2K	BMP2 inducible kinase [Source:HGNC Symbol;Acc:HGNC:18041]	-1.64	-0.04	-0.98	-0.11	-0.84	-0.54	**0.54**

84957	RELT	RELT, TNF receptor [Source:HGNC Symbol;Acc:HGNC:13764]	-0.54	-0.54	-0.74	-0.35	-0.54	-0.55	**0.54**
154810	AMOTL1	angiomotin like 1 [Source:HGNC Symbol;Acc:HGNC:17811]	-1.56	-0.26	-0.87	-0.22	-0.91	-0.54	**0.54**
3607	FOXK2	forkhead box K2 [Source:HGNC Symbol;Acc:HGNC:6036]	-0.36	-1.25	-0.85	-0.23	-0.80	-0.54	**0.54**
200844	C3orf67	chromosome 3 open reading frame 67 [Source:HGNC Symbol;Acc:H	-0.57	-0.71	-0.91	-0.17	-0.64	-0.54	**0.54**
2030	SLC29A1	solute carrier family 29 member 1 (Augustine blood group) [Source	-1.12	-0.08	-0.72	-0.36	-0.60	-0.54	**0.54**
494143	CHAC2	ChaC cation transport regulator homolog 2 [Source:HGNC Symbol;A	-0.38	-0.70	-0.47	-0.63	-0.54	-0.55	**0.54**
57482	KIAA1211	KIAA1211 [Source:HGNC Symbol;Acc:HGNC:29219]	-0.73	-0.34	-0.98	-0.18	-0.54	-0.58	**0.54**
1633	DCK	deoxycytidine kinase [Source:HGNC Symbol;Acc:HGNC:2704]	-0.89	-0.33	-0.58	-0.49	-0.61	-0.54	**0.54**
64208	POPDC3	popeye domain containing 3 [Source:HGNC Symbol;Acc:HGNC:176	-0.95	-0.76	-0.93	-0.14	-0.86	-0.54	**0.54**
7738	ZNF184	zinc finger protein 184 [Source:HGNC Symbol;Acc:HGNC:12975]	-0.60	-0.48	-0.74	-0.53	-0.54	-0.64	**0.54**
1786	DNMT1	DNA methyltransferase 1 [Source:HGNC Symbol;Acc:HGNC:2976]	-0.60	-0.47	-1.66	-0.53	-0.54	-1.10	**0.54**
55165	CEP55	centrosomal protein 55 [Source:HGNC Symbol;Acc:HGNC:1161]	-0.80	-0.27	-1.48	-0.63	-0.54	-1.05	**0.54**
3992	FADS1	fatty acid desaturase 1 [Source:HGNC Symbol;Acc:HGNC:3574]	-0.79	-0.29	-0.98	-0.09	-0.54	-0.54	**0.54**
8971	H1FX	H1 histone family member X [Source:HGNC Symbol;Acc:HGNC:472	-0.90	-0.43	-0.71	-0.36	-0.67	-0.54	**0.54**
6874	TAF4	TATA-box binding protein associated factor 4 [Source:HGNC Symbo	-0.89	-0.93	-0.71	-0.36	-0.91	-0.53	**0.53**
29090	TIMM21	translocase of inner mitochondrial membrane 21 [Source:HGNC Sy	-1.20	-0.52	-0.62	-0.44	-0.86	-0.53	**0.53**
8975	USP13	ubiquitin specific peptidase 13 [Source:HGNC Symbol;Acc:HGNC:12	-0.80	-0.29	-0.78	-0.28	-0.54	-0.53	**0.53**
3315	HSPB1	heat shock protein family B (small) member 1 [Source:HGNC Symbo	-0.79	-0.27	-1.18	-0.35	-0.53	-0.76	**0.53**
9942	XYLB	xylulokinase [Source:HGNC Symbol;Acc:HGNC:12839]	-0.80	-1.02	-0.37	-0.69	-0.91	-0.53	**0.53**
5558	PRIM2	DNA primase subunit 2 [Source:HGNC Symbol;Acc:HGNC:9370]	-0.49	-0.56	-0.83	-0.64	-0.53	-0.74	**0.53**
145508	CEP128	centrosomal protein 128 [Source:HGNC Symbol;Acc:HGNC:20359]	-0.54	-0.52	-1.48	-0.62	-0.53	-1.05	**0.53**
254428	SLC41A1	solute carrier family 41 member 1 [Source:HGNC Symbol;Acc:HGN	-0.49	-0.66	-0.80	-0.25	-0.58	-0.53	**0.53**
6839	SUV39H1	suppressor of variegation 3-9 homolog 1 [Source:HGNC Symbol;Ac	-0.69	-0.36	-1.36	-0.54	-0.53	-0.95	**0.53**
6722	SRF	serum response factor [Source:HGNC Symbol;Acc:HGNC:11291]	-1.89	-0.87	-0.65	-0.39	-1.38	-0.52	**0.52**
10528	NOP56	NOP56 ribonucleoprotein [Source:HGNC Symbol;Acc:HGNC:15911]	-0.83	-0.70	-0.70	-0.34	-0.77	-0.52	**0.52**
9031	BAZ1B	bromodomain adjacent to zinc finger domain 1B [Source:HGNC Syn	-1.27	-0.33	-0.66	-0.38	-0.80	-0.52	**0.52**
10541	ANP32B	acidic nuclear phosphoprotein 32 family member B [Source:HGNC S	-1.32	-0.43	-0.71	-0.33	-0.87	-0.52	**0.52**
3654	IRAK1	interleukin 1 receptor associated kinase 1 [Source:HGNC Symbol;A	-0.73	-0.55	-0.56	-0.48	-0.64	-0.52	**0.52**
5198	PFAS	phosphoribosylformylglycinamidine synthase [Source:HGNC Symbo	-1.67	-0.78	-0.83	-0.21	-1.23	-0.52	**0.52**
10248	POP7	POP7 homolog, ribonuclease P/MRP subunit [Source:HGNC Symbol	-0.72	-0.32	-0.70	-0.48	-0.52	-0.59	**0.52**
55095	SAMD4B	sterile alpha motif domain containing 4B [Source:HGNC Symbol;Ac	-0.96	-0.07	-0.66	-0.83	-0.52	-0.75	**0.52**
9734	HDAC9	histone deacetylase 9 [Source:HGNC Symbol;Acc:HGNC:14065]	-1.04	-0.16	-0.84	-0.20	-0.60	-0.52	**0.52**
262	AMD1	adenosylmethionine decarboxylase 1 [Source:HGNC Symbol;Acc:H	-0.57	-0.47	-0.73	-0.31	-0.52	-0.52	**0.52**
27301	APEX2	apurinic/apyrimidinic endodeoxyribonuclease 2 [Source:HGNC Sym	-0.39	-0.64	-0.74	-0.51	-0.52	-0.62	**0.52**
10915	TCERG1	transcription elongation regulator 1 [Source:HGNC Symbol;Acc:HG	-0.84	-0.46	-0.71	-0.33	-0.65	-0.52	**0.52**
10526	IPO8	importin 8 [Source:HGNC Symbol;Acc:HGNC:9853]	-0.67	-0.36	-1.22	-0.27	-0.52	-0.74	**0.52**
55319	TMA16	translation machinery associated 16 homolog [Source:HGNC Symbo	-0.38	-0.65	-1.02	-0.20	-0.52	-0.61	**0.52**
50628	GEMIN4	gem nuclear organelle associated protein 4 [Source:HGNC Symbol;	-0.98	-1.15	-0.71	-0.32	-1.06	-0.51	**0.51**
4001	LMNB1	lamin B1 [Source:HGNC Symbol;Acc:HGNC:6637]	-0.59	-0.43	-1.36	-0.65	-0.51	-1.00	**0.51**
899	CCNF	cyclin F [Source:HGNC Symbol;Acc:HGNC:1591]	-0.74	-0.29	-1.26	-0.45	-0.51	-0.86	**0.51**
316	AOX1	aldehyde oxidase 1 [Source:HGNC Symbol;Acc:HGNC:553]	-1.36	-1.16	-0.56	-0.47	-1.26	-0.51	**0.51**
78995	C17orf53	chromosome 17 open reading frame 53 [Source:HGNC Symbol;Acc:	-0.65	-0.38	-0.58	-0.45	-0.52	-0.51	**0.51**
23109	DDN	dendrin [Source:HGNC Symbol;Acc:HGNC:24458]	-2.33	-0.48	-0.61	-0.42	-1.41	-0.51	**0.51**
586	BCAT1	branched chain amino acid transaminase 1 [Source:HGNC Symbol;A	-1.27	-1.21	-0.90	-0.12	-1.24	-0.51	**0.51**
995	CDC25C	cell division cycle 25C [Source:HGNC Symbol;Acc:HGNC:1727]	-0.32	-0.71	-1.87	-0.57	-0.51	-1.22	**0.51**
57696	DDX55	DEAD-box helicase 55 [Source:HGNC Symbol;Acc:HGNC:20085]	-0.49	-0.53	-0.63	-0.41	-0.51	-0.52	**0.51**
3192	HNRNPU	heterogeneous nuclear ribonucleoprotein U [Source:HGNC Symbol	-0.93	-0.21	-0.61	-0.41	-0.57	-0.51	**0.51**
9133	CCNB2	cyclin B2 [Source:HGNC Symbol;Acc:HGNC:1580]	-0.97	-0.04	-1.21	-0.35	-0.51	-0.78	**0.51**
6835	SURF2	surfeit 2 [Source:HGNC Symbol;Acc:HGNC:11475]	-0.69	-0.70	-0.52	-0.50	-0.69	-0.51	**0.51**
84733	CBX2	chromobox 2 [Source:HGNC Symbol;Acc:HGNC:1552]	-0.89	-0.13	-0.42	-0.65	-0.51	-0.53	**0.51**
150468	CKAP2L	cytoskeleton associated protein 2 like [Source:HGNC Symbol;Acc:H	-0.79	-0.23	-1.84	-0.71	-0.51	-1.27	**0.51**
7533	YWHAH	tyrosine 3-monooxygenase/tryptophan 5-monooxygenase activatic	-1.33	-0.03	-0.52	-0.50	-0.68	-0.51	**0.51**
10534	SSSCA1	Sjogren syndrome/scleroderma autoantigen 1 [Source:HGNC Symb	-0.42	-0.62	-0.68	-0.33	-0.52	-0.51	**0.51**
51203	NUSAP1	nucleolar and spindle associated protein 1 [Source:HGNC Symbol;A	-0.44	-0.57	-1.62	-0.80	-0.51	-1.21	**0.51**
10882	C1QL1	complement C1q like 1 [Source:HGNC Symbol;Acc:HGNC:24182]	-1.04	-0.47	-0.31	-0.70	-0.76	-0.51	**0.51**
286151	FBXO43	F-box protein 43 [Source:HGNC Symbol;Acc:HGNC:28521]	-0.34	-0.67	-1.51	-0.59	-0.50	-1.05	**0.50**
3005	H1F0	H1 histone family member 0 [Source:HGNC Symbol;Acc:HGNC:471	-1.25	-0.29	-0.58	-0.42	-0.77	-0.50	**0.50**
22809	ATF5	activating transcription factor 5 [Source:HGNC Symbol;Acc:H	-0.83	-0.99	-0.41	-0.59	-0.91	-0.50	**0.50**
115004	CGAS	cyclic GMP-AMP synthase [Source:HGNC Symbol;Acc:HGNC:21367]	-0.40	-0.61	-0.72	-0.51	-0.50	-0.61	**0.50**
23511	NUP188	nucleoporin 188 [Source:HGNC Symbol;Acc:HGNC:17859]	-0.73	-0.33	-0.59	-0.41	-0.53	-0.50	**0.50**
80764	THAP7	THAP domain containing 7 [Source:HGNC Symbol;Acc:HGNC:23190	-0.43	-0.57	-0.99	-0.41	-0.50	-0.70	**0.50**
51592	TRIM33	tripartite motif containing 33 [Source:HGNC Symbol;Acc:HGNC:162	-0.73	-0.27	-0.66	-0.71	-0.50	-0.69	**0.50**

* Top 50 genes, in bold type, are shown in Figure 2.

** Gene list sorted according to "Abs(Overlap)_logFC": the absolute value of shMYC:shTip60 overlap (in log2)
